# EZH2-mediated Epigenetic Silencing of miR-29/miR-30 targets LOXL4 and contributes to Tumorigenesis, Metastasis, and Immune Microenvironment Remodeling in Breast Cancer

**DOI:** 10.7150/thno.44849

**Published:** 2020-07-09

**Authors:** Huilong Yin, Yidi Wang, Ye Wu, Xiang Zhang, Xiaofang Zhang, Jun Liu, Ting Wang, Jing Fan, Jianyong Sun, Angang Yang, Rui Zhang

**Affiliations:** 1 Henan Key Laboratory of Immunology and Targeted Therapy, School of Laboratory Medicine, Xinxiang Medical University, Xinxiang, Henan 453003, China.; 2 The State Key Laboratory of Cancer Biology, Department of Immunology, Fourth Military Medical University, Xi'an, Shaanxi 710032, China.; 3 The State Key Laboratory of Cancer Biology, Department of Biochemistry and Molecular Biology, Fourth Military Medical University, Xi'an, Shaanxi 710032, China.; 4 Department of Thyroid, Breast and Vascular Surgery, Xijing Hospital, Fourth Military Medical University, Xi'an, China.; 5 Henan Collaborative Innovation Center of Molecular Diagnosis and Laboratory Medicine, School of Laboratory Medicine, Xinxiang Medical University, Xinxiang, Henan 453003, China.; 6 Department of Thoracic Surgery, Tangdu hospital, Fourth Military Medical University, Xi'an, China.

**Keywords:** Breast cancer, EZH2, LOXL4, miR-29b/miR-30d, Macrophage

## Abstract

Enhancer of Zeste Homolog 2 (EZH2), a key epigenetic regulator, is involved in breast cancer progression and metastasis. LOXL4 is increasingly recognized as an important player in cancer progression. To date, how EZH2 regulates LOXL4 in the progression of breast cancer remains unclear.

**Methods:** We evaluated the association between LOX family proteins and EZH2 in invasive breast carcinoma through the starBase v2.0 analysis, and its correlation with breast tumorigenesis using the Oncomine dataset. We then applied miRcode data combined with gene expression omnibus (GEO) data to screen candidate miRNAs mediating the regulation of LOXL4 by EZH2. We explored the regulatory mechanism of EZH2, miR-29b/miR-30d, and LOXL4 in breast cancer cells by qRT-PCR, Western blotting, cell proliferation, colony formation, and wound healing assays, xenograft experiments, dual-luciferase reporter assay, and chromatin immunoprecipitation. All statistical tests were two-sided.

**Results:** Inhibition of EZH2 or LOXL4, or miR-29b/miR-30d overexpression, decreased breast cancer cell proliferation, migration, and metastasis *in vitro* and *in vivo*. LOXL4 was identified as a direct target of miR-29b and miR-30d. EZH2 inhibition enhanced miR-30d and miR-29b transcription via promoter binding activity, leading to the reduced expression of LOXL4. Immunohistochemical analysis of human breast cancer specimens and flow cytometry analysis of tumor-infiltrating macrophages in mice showed a positive association of EZH2 with LOXL4 expression and macrophage infiltration.

**Conclusions:** Our findings identified EZH2-miR-29b/miR-30d-LOXL4 signaling pathway was involved in breast tumorigenesis, and suggested that the epigenetic modulation represents a potential therapeutic target for breast cancer by controlling macrophage activation.

## Introduction

Breast cancer is the most frequent malignancy among women. Most breast cancer-related deaths are associated with the spread of malignant cells from a primary tumor to distant sites in the body, resulting in the growth of new tumors in other organs 1. Although early breast cancer can be treated with good long-term results, metastasis is exceedingly difficult to prevent and manage clinically. Thus, it is critical to better understand the molecular mechanisms underlying metastasis to enable the development of strategies for advanced breast cancer.

Enhancer of Zeste homolog 2 (EZH2) is a core protein of the polycomb-repressive complex 2 (PRC2) that catalyzes dimethylation and trimethylation of Lys 27 of histone H3, a repressive chromatin mark. EZH2 is associated with malignant transformation and is reported to facilitate cancer cell proliferation, metastasis, and cancer stem cell expansion 2-9. Furthermore, EZH2 has been identified as an oncogene, especially in prostate and breast cancers, by epigenetically impeding the expression of various tumor suppressor genes 10-12. Thus, EZH2 is considered a promising therapeutic target 13. Currently, several inhibitors targeting EZH2 are under investigation for cancer therapy 14, 15. However, the role of EZH2 as a driving force in breast cancer progression still needs to be characterized.

The extracellular matrix (ECM) is a necessary component of tissues in multicellular organisms. Components of the ECM include collagens, laminins, fibronectin, glycosaminoglycans and proteoglycans, matricellular proteins, and ECM remodeling enzymes that act as a structural scaffold and maintain the architecture and homeostasis of tissues 16, 17. However, the ECM is much more than a common structural framework as it can affect cell fate via multiple mechanisms 18. Under pathological conditions, significant changes in ECM composition are generally regarded as one of the greatest extrinsic drivers of cancer progression 19. At the primary site, cancer cells can change the ECM structure directly or indirectly through recruitment and activation of stromal cells 20. To metastasize, tumor cells release specific factors that alter the ECM structure at secondary sites to create a permissive microenvironment required for subsequent colonization of disseminated cancer cells 21.

It has been reported that changes in interstitial ECM mechanics promote breast tumorigenesis 22, and ECM remodeling contributes to breast cancer cell invasion 23. Lysyl oxidase (LOX) family proteins that mediate the cross-linking of collagen and elastin, two basic components of the ECM, are considered key regulators in remodeling of the cancer-associated ECM and premetastatic niche formation 24. The LOX family includes five members: LOX and LOX-like 1 to 4 (LOXL1, LOXL2, LOXL3, and LOXL4). The family shares a highly conserved C-terminal domain that contains a copper-binding site for lysine tryosylquinone and a cytokine receptor-like region for carbonyl cofactor formation. LOXL4, considered to be a necessary protein for cartilage maturation and cartilage ECM deposition 25, is widely expressed in various tissues, especially in endothelial cells 26. Also, transforming growth factor-beta 1-activated LOXL4 promotes vascular ECM remodeling 27.

Recent studies have described the involvement of LOXL4 in cancer progression, with reports of both oncogenic and tumor-suppressive functions. LOXL4 serves as a tumor suppressor in human bladder cancer and lung cancer 28, 29, whereas it promotes proliferation and/or metastasis of gastric cancer, head and neck squamous cell carcinoma, and hepatocellular carcinoma 30-32. We speculated that the progressive or repressive roles of LOXL4 in different tumors depend on the cellular context. Currently, the functions of LOXL4 regulated by EZH2 in breast cancer are not understood.

Here, we showed that EZH2 promoted the expression of LOXL4. We further demonstrated that EZH2-mediated epigenetic silencing of miR-29b or miR-30d reprogrammed the expression of LOXL4, which influenced cell proliferation and metastasis in the progression of breast cancer. Our study identified an important transcriptional axis comprised of EZH2, miR-29b/miR-30d, and LOXL4, which participated in driving the development of breast cancer by regulating macrophage activation.

## Methods

### Clinical specimens

Frozen and paraffin-embedded primary breast cancer tissues and corresponding adjacent nontumorous breast samples were obtained from Chinese patients at Xijing Hospital (Xi'an, China), a subsidiary hospital of the Fourth Military Medical University. The use of clinical specimens in this study was approved by the Animal Experiment Administration Commission in Fourth Military Medical University. The clinicopathological characteristics of breast cancer patients are summarized in Table S1.

### EZH2 inhibitors and cell lines

EZH2 inhibitors (DZNep, GSK343, EPZ005687, and UNC1999) were obtained from Selleck. The cell lines MDA-MB-231, MCF-7, and 4T1 were obtained from the American Type Cell Culture Collection (ATCC). All cell lines were cultured in Dulbecco's Modified Eagle's Medium (DMEM, Gibco) supplemented with 10% fetal bovine serum (FBS, Gibco). The cells were incubated at 37 °C with 5% CO_2_.

### Transfection and lentiviral transduction

For siRNA, miRNA mimic, and miRNA inhibitor transfection, cells were seeded one day before transfection. The transfection was performed when they were at about 70% confluency using Lipofectamine RNAiMAX (Invitrogen) according to the manufacturer's instructions. The siRNA duplex oligonucleotides used are listed in Table S2. Lipofectamine 3000 (Invitrogen) was used for plasmid transfections following the manufacturer's instructions.

For lentiviral transduction, the packaging of lentivirus was performed using a transient co-transfection system of HEK-293T cells in 60-mm dishes with 1 µg pMD2G, 3 µg psPAX2, and 4 µg PCDH or PCDH-miR-29b*/*miR-30d*/*LOXL4. The cloning primers for generating these plasmids are listed in Table S3. After 48 h and 72 h, the culture supernatant was collected and filtered. For lentiviral transduction of MDA-MB-231 cells, MDA-MB-231 cells were plated at a density of 3 × 10^4^ cells per well in 6-well plates and cultured overnight. The medium was replaced with the infection medium containing 1 mL fresh medium, 1 mL lentiviral supernatant and 20 µg polybrene (Sigma) to assist the uptake of viral particles. After infection, cells were cultured in the medium containing puromycin (1 µg*/*mL) for selection and analyzed by qRT-PCR and immunoblotting.

### Quantitative real-time PCR (qRT-PCR)

Total RNA, including miRNA, was isolated from cells using Trizol (Invitrogen). For qRT-PCR analysis of miRNAs, total RNA was isolated using Trizol and reverse transcribed to cDNA using the RT2 miRNA First-Strand kit (Qiagen) according to the manufacturer's instructions. Subsequently, quantitative PCR was performed using miR-29b or miR-30d specific primers. U6 was used as a normalizing control for miRNA qRT-PCR analysis. For qRT-PCR analysis of mRNAs, the first-strand cDNA was synthesized using a PrimeScript qRT-PCR kit (Takara) with specific primers. The primers used for qRT-PCR are listed in Table S4.

### Immunoblotting

Proteins were separated by SDS/PAGE gels and transferred onto nitrocellulose membranes (Millipore). The membranes were blocked in TBST (10 mM Tris-HCl pH 7.4, 150 mM NaCl, and 0.1% Tween-20) containing 5% non-fat milk at room temperature for 60 min. Primary antibodies were diluted in TBST containing 5% BSA, and used at the following concentrations: rabbit anti-EZH2 (1:1000, 5246, Cell Signaling Technology), rabbit anti-H3K27me3 (1:1000, 9733, Cell Signaling Technology), rabbit anti-Dicer1 (1:1000, 5362, Cell Signaling Technology), rabbit anti-LOXL4 (1:1000, 88186, Abcam), mouse anti-β-actin (1:5000, Sigma). The membranes were incubated with primary antibodies at 4 °C overnight, then washed three times with TBST and incubated with HRP-conjugated anti-mouse IgG (1:10,000, 7076, Cell Signaling Technology) or anti-rabbit IgG (1:10,000, 7074, Cell Signaling Technology) diluted in TBST containing 1% non-fat milk at room temperature for 60 min. After final washing with TBST, the membranes were developed by using ECL and visualized using Tanon 5500.

### Cell viability assay

Cells were seeded in 96-wells plate with 1×10^3^ cells per well. Cell Counting Kit-8 (CCK-8) assays were carried out every day. In brief, cells were cultured in 100 μL culture medium containing 10 µL Cell Counting Kit-8 (0.5 mg/mL) reagent at 37 °C for 1-4 h. The absorbance was measured by BIO-RAD Microplate Reader at 450 nm. The experiment was performed in triplicate wells for at least three times.

### Wound healing assay

For wound healing assays, breast cancer cell lines were used when the cells reached 90% confluency. Then the monolayer cells were scratched and cultured under normal conditions. Wound closure was recorded by taking photographs under a microscope.

### Colony formation assay

Cells were seeded at the density of 1000 cells/well in 6-well plates and cultured at 37 °C for colony formation. After 7-14 days, the cells were fixed with 4% paraformaldehyde for 15 min and stained with 0.5% (w/v) crystal violet (Sigma) for 20 min at room temperature. The colonies were photographed using a scanner (Odyssey) and counted by image J software.

### Dual-luciferase reporter assay

Cells were seeded in 48-well plates one day before transfection. 5 ng of plasmids encoding Renilla luciferase and 100 ng of plasmids containing firefly luciferase reporters were transfected using Lipofectamine Reagent 3000 (Invitrogen) when the cells were 70-80% confluent. The activity of luciferase was tested using the Dual-Luciferase Reporter Assay System (Promega). Firefly luciferase values were normalized to internal Renilla luciferase values.

### Chromatin immunoprecipitation (ChIP) assay

ChIP analysis of EZH/H3K27me3 was performed using the SimpleChIP Enzymatic Chromatin IP Kit (Cell Signaling Technology) according to manufacturer's instructions. Primer sequences for ChIP analysis are listed in Table S4.

### Immunohistochemistry (IHC)

Standard IHC staining was performed as previously described 33. In brief, after de-paraffinization and rehydration, sections were subjected to heat-induced antigen unmasking. Slides were blocked with 5% goat serum and then incubated with various primary antibodies at 4 °C overnight. The UltraView Universal DAB detection kit (Ventana Medical Systems) was used for color development of the slides. Images were captured using a microscope (Olympus) and processed with identical settings.

### Flow cytometry

Tumors were excised from mice, minced, and digested with 10 U/mL collagenase I (Gibco) and 30 U/mL DNase I in RPMI medium for 90 min at 37 °C and filtered through a 40 μm nylon filter (BD Biosciences) to obtain single-cell suspensions. After lysis of the red blood cells, the remaining cells were washed with complete RPMI medium and stained with the surface antibody: F4/80-PE (T45-2342) from BD Bioscience from eBioscience. For intracellular staining, cells were fixed and permeated with Fixation and Permeabilization Solution (BD Biosciences), washed three times, and stained with NOS2-APC (CXNFT) and CD206-Alexa Fluor 488 (MR6F3) from eBioscience for 0.5 h in the dark at 4 °C, then subjected to flow cytometry. Data were analyzed with FlowJo software.

### Xenograft tumor model

Six-week-old BALB/c or BALB/c nude female mice were purchased from the Model Animal Research Center of Nanjing University (Nanjing, China). All animal procedures were carried out according to the criteria outlined in the Guide for the Care and Use of Laboratory Animals prepared by the National Academy of Sciences and published by the National Institutes of Health (Bethesda, MD). MDA-MB-231 or 4T1 cells were treated accordingly and injected subcutaneously into the right flank of each mouse (5×10^6^ cells/mouse). Tumor volume was monitored and calculated by using the following formula: *V* (mm3) = *a* × *b*^2^/2, in which a and b mean the long and short diameters, respectively. The tail-vein injection assays were performed as previously described 34. In brief, MDA-MB-231 cells stably expressing luciferase were subjected to various treatments and injected into the BALB/c nude mice via the tail-vein (5 × 10^5^ cells/mouse). Metastatic foci were detected by bioluminescence imaging. Mice were first injected with luciferin (300 mg/kg) prior to imaging, anesthetized with 2.5% isoflurane, and then imaged in an IVIS Spectrum Imaging System (Caliper, USA). Images were analyzed with Living Image software (Caliper, USA). Bioluminescent flux was determined for the tumors.

For LOXL4 inhibition, mice were i.p. injected with β-aminoproprionitrile (BAPN) (100 mg/kg, Sigma) dissolved in PBS and administered in 200 μL volume on day -1 and then daily till the mice were sacrificed. For EZH2 inhibition, mice were inoculated subcutaneously in the left flank with 4T1 cells in 100 μL DMEM. After the detection of tumors, mice were treated with 25 mg/kg intraperitoneal UNC1999 twice a week. A control group received intraperitoneal vehicle (5% DMSO in corn oil).

### Statistical analysis

Statistical significance was determined by two-tailed t-test, and differences were considered statistically significant when *P* < 0.05 (*), *P* < 0.01 (**) or *P* < 0.001 (***).

## Results

### EZH2 promotes LOXL4 expression in breast cancer cells

**Figure 1:**
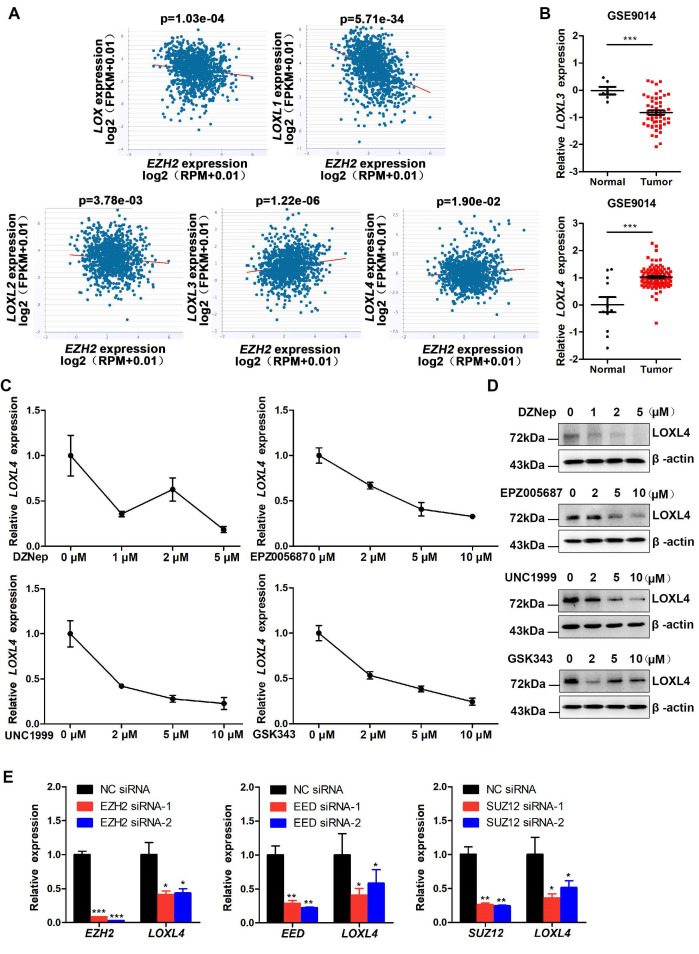
** EZH2 promotes LOXL4 expression in breast cancer cells. (A)** Correlation analysis of LOX, LOXL1, LOXL2, LOXL3, LOXL4, and EZH2 in invasive breast carcinoma using starBase v2.0 (http://starbase.sysu.edu.cn/). **(B)** mRNA expression level of LOXL3 and LOXL4 in normal and tumor breast tissues using the GSE9014 dataset.** (C)** qRT-PCR analysis of LOXL4 in MDA-MB-231 cells in response to different concentrations of DZNep, UNC1999, EPZ005687, or GSK343. **(D)** Western blotting analysis of LOXL4 expression in MDA-MB-231 cells exposed to different concentrations of DZNep, UNC1999, EPZ005687, or GSK343.** (E)** Knock-down of EZH2, EED, and SUZ12 by siRNAs reduced LOXL4 gene expression in MDA-MB-231 cells.

EZH2 has been identified as an oncogene in breast cancer that functions by epigenetically inhibiting the expression of various tumor suppressor genes. ECM remodeling is considered as one of the significant extrinsic drivers of tumor progression. It remains unclear how EZH2 regulates ECM remodeling in the progression of breast cancer. We investigated the mechanisms involved in the regulation of ECM remodeling by EZH2 by performing starBase v2.0 analysis to evaluate the correlation between EZH2 and LOX family proteins, which play a critical role in the formation and repair of the ECM in invasive breast carcinoma. We detected an inverse correlation of EZH2 with LOX, LOXL1, and LOXL2 but a positive correlation with LOXL3 and LOXL4 at the mRNA level (Figure 1A). Further analysis of data derived from the GSE9014 set 35 showed that the expression of LOXL4, but not LOXL3, was significantly higher in breast cancer samples than in normal breast samples (Figure 1B). The results implied that EZH2 might regulate the progression of breast cancer through LOXL4.

We investigated the roles of EZH2 and LOXL4 in breast cancer by treating MDA-MB-231 cells with numerous EZH2 inhibitors, such as DZNep, GSK343, UNC1999, or EPZ005687. As shown in Figure S1A, a significant decrease in H3K27me3 was observed after treatment with EZH2 inhibitors. Colony formation and CCK-8 assays showed that cell proliferation was significantly impaired in breast cancer cells by EZH2 inhibitor treatment (Figure S1B-C). Next, we measured LOXL4 expression in EZH2 inhibitor-treated cells. As displayed in Figure 1C-D, LOXL4 expression at both mRNA and protein levels was remarkably decreased in EZH2 inhibitor-treated MDA-MB-231 cells compared with control cells. Similar results were obtained in 4T1 mouse breast cancer cells (Figure S2). Cell viability was also significantly inhibited in EZH2 knock-down cells compared with control cells (Figure S3A-C), consistent with the observation of EZH2 inhibitors treatment.

The inhibition of EZH2 activity by EZH2 knock-down resulted in reduced LOXL4 expression (Figure 1E; Figure S3D). Furthermore, we found that knock-down of PRC2 complex subunits SUZ12 and EED also resulted in reduced expression of LOXL4 (Figure 1E), indicating that EZH2 affected LOXL4 expression in a PRC2-dependent manner. Increased expression of LOXL1 and decreased expression of LOXL3 were observed upon EZH2 knock-down (Figure S3E), suggesting that the expression of other LOX family members was also affected by EZH2. The promoter activity results demonstrated that LOXL4 was not transcriptionally regulated directly by EZH2 (Figure S3F). Collectively, these data suggested that EZH2 was a critical regulator of LOXL4 in breast cancer.

### LOXL4 promotes cell proliferation and migration *in vitro*

**Figure 2:**
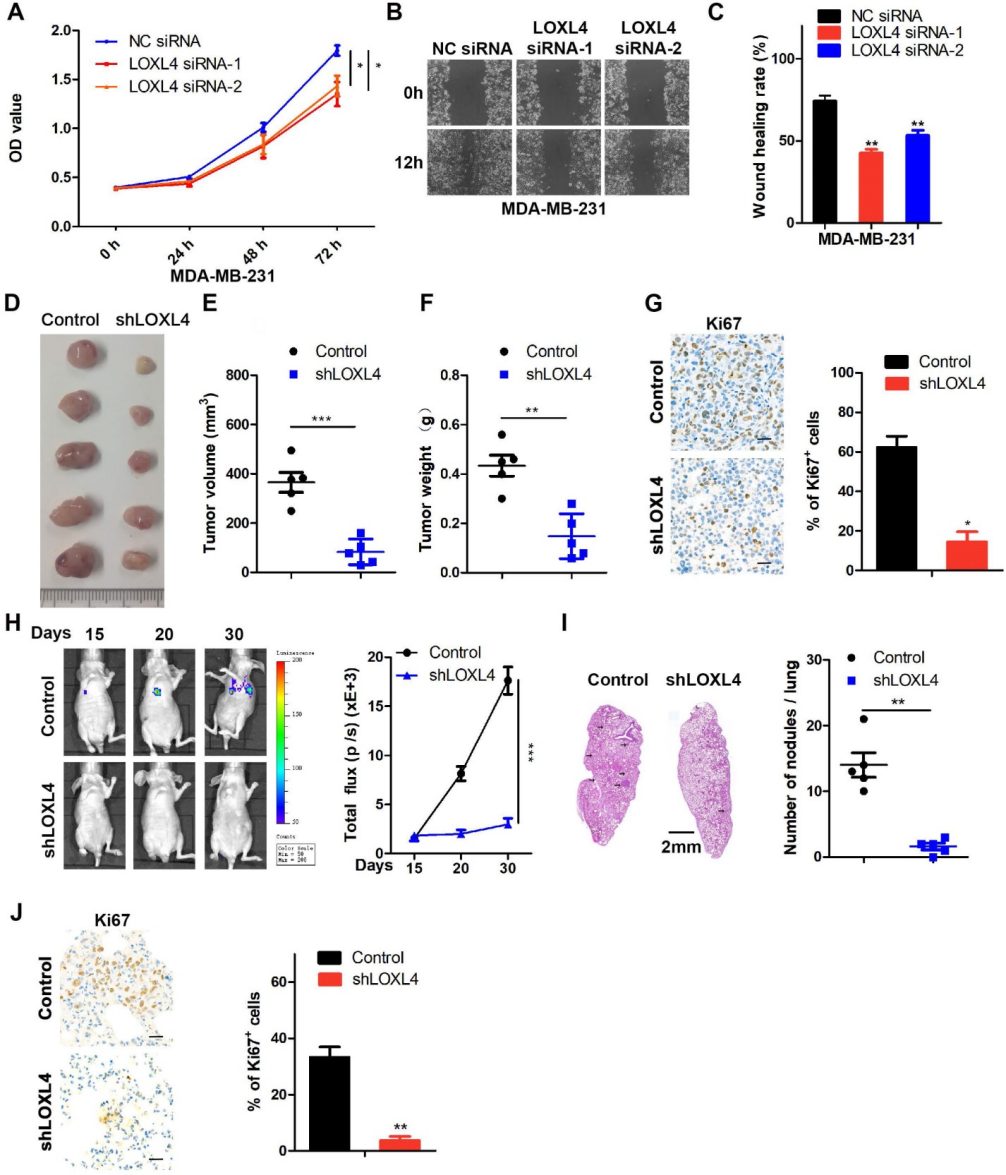
** LOXL4 knock-down inhibits cell proliferation and migration *in vitro* and tumor metastasis *in vivo.* (A)** Cell proliferation analysis was performed in MDA-MB-231 cells transfected with NC siRNA or LOXL4 siRNA.** (B and C)** Representative images of scratch wound healing assays in MDA-MB-231 cells transfected with NC siRNA or LOXL4 siRNA (B). Quantification of wound healing rates (C). **(D)** Representative images of tumors in nude mice subcutaneously inoculated with control shRNA- or LOXL4 shRNA-treated MDA-MB-231 cells. **(E and F)** The tumor volume (E) and weight (F) in nude mice subcutaneously inoculated with control shRNA- or LOXL4 shRNA-treated MDA-MB-231 cells. **(G)** Staining of proliferation marker Ki67 in tumor tissues from nude mice (Left panel). Quantification of Ki67 expression (right panel). Scale bar: 30 µm. **(H)** Nude mice were injected with tumor cells through the tail vein, and the representative bioluminescent images are shown at indicated time points (left panel). Total photon flux at the indicated times is shown in the right panel. **(I)** Representative HE staining pictures of lung sections of control and LOXL4-depleted group. Arrows indicate metastatic nodules (left panel). Quantification of metastatic lung nodules (right panel). **(J)** Representative human Ki67 staining pictures of lung sections in control and LOXL4 shRNA group (left panel). Quantification of Ki67 expression (right panel). Scale bar: 30 µm.

The observations described above prompted us to explore the functional role of LOXL4 in breast tumorigenesis and metastasis. LOXL4 siRNAs were transfected into MDA-MB-231 and MCF-7 cells, resulting in a drastically decreased expression of LOXL4 as confirmed by Western blotting (Figure S4A-B). The LOXL4 siRNA efficiency in 4T1 cells was validated by qRT-PCR since the LOXL4 antibody could not detect the mouse LOXL4 protein (Figure S4C). As shown in Figure 2A-C and Figure S4D-E, various cellular analyses showed that the proliferation and migration of breast cancer cells were significantly impaired by LOXL4 knock-down.

Stable cell lines were generated by introducing vehicle (Control) or LOXL4 shRNA into MDA-MB-231 cells that significantly decreased LOXL4 expression at both mRNA and protein levels (Figure S4F-G). These cell lines were used to evaluate the effects of LOXL4 on tumor growth *in vitro* and* in vivo*. As determined by various *in vitro* experiments, such as CCK-8 (Figure S4H), colony formation (Figure S4I), and wound healing (Figure S4J) assays, proliferation, and migration of MDA-MB-231 cells expressing LOXL4 shRNA were significantly impaired.

### LOXL4 promotes tumor metastasis* in vivo*

MDA-MB-231 cells stably expressing LOXL4 shRNA or control cells were injected subcutaneously into the flanks of nude mice. As is evident from Figure 2D-F, LOXL4 knock-down significantly inhibited breast tumorigenesis *in vivo*. Tumors formed from LOXL4 shRNA-transfected MDA-MB-231 cells exhibited decreased positivity for Ki67 than those from control cells (Figure 2G)*.* Furthermore, we examined the role of LOXL4 in breast cancer metastasis *in vivo* by intravenous injection of the luciferase-labeled MDA-MB-231-control or -shLOXL4 cells into nude mice. We found that control tumors grew profoundly bigger than the shLOXL4 tumors (Figure 2H). More tumor nodules and increased Ki67 staining were found in the lungs of the control group compared with the -shLOXL4 group, indicating that LOXL4 promoted growth and lung metastasis of MDA-MB-231 cells (Figure 2I-J).

**Figure 3:**
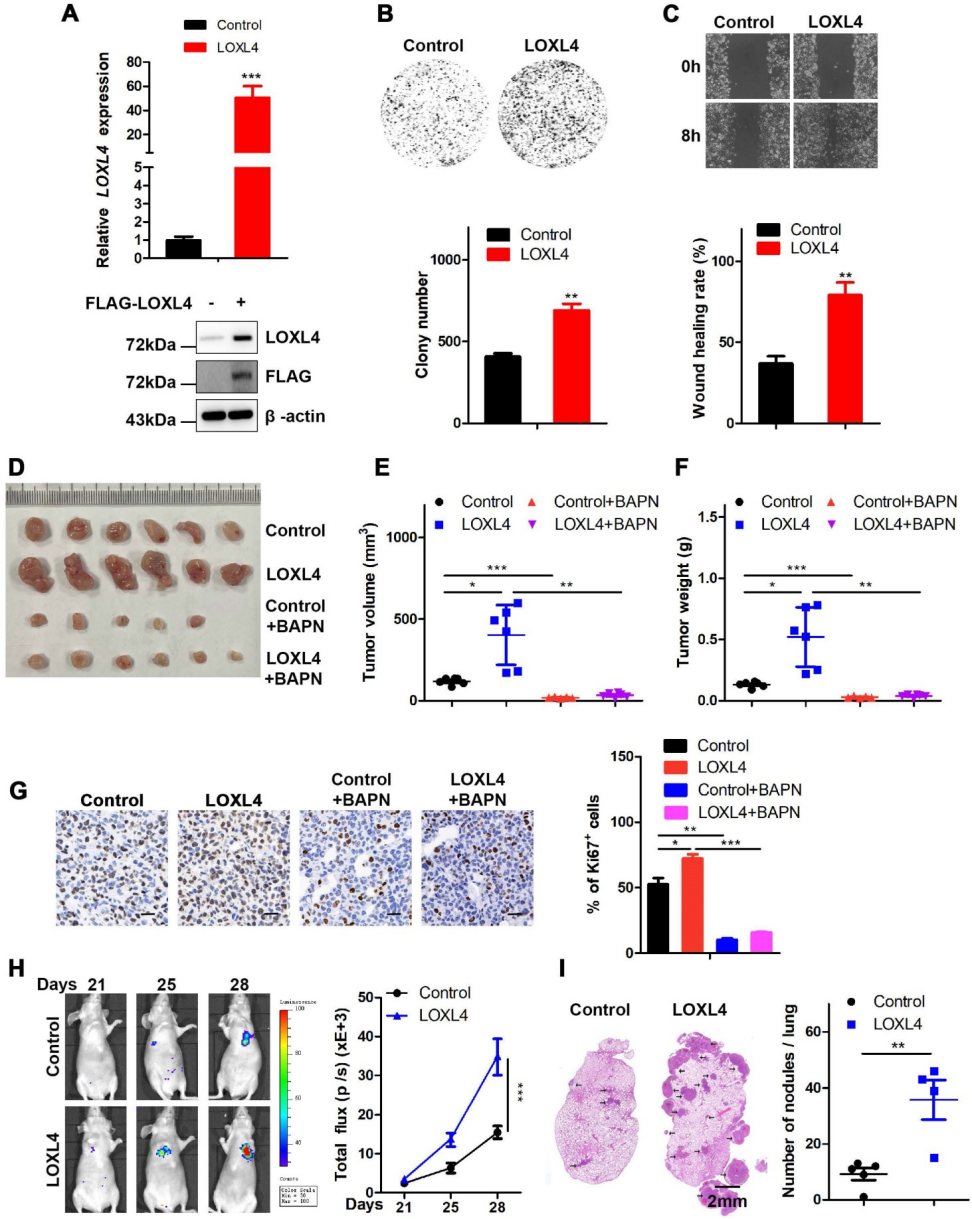
** Overexpression of LOXL4 promotes cell proliferation and migration *in vitro* and tumor metastasis *in vivo*. (A)** qRT-PCR and Western blotting analysis of LOXL4 expression in MDA-MB-231 cells transfected with PCDH or PCDH-LOXL4. **(B)** Colony formation assays of MDA-MB-231 cells transfected with PCDH or PCDH-LOXL4 (upper panel). Quantification of Colony formation assays is shown (lower panel).** (C)** Representative images of scratch wound healing assays of MDA-MB-231 cells stably expressing LOXL4 (upper panel). Quantification of wound healing rates (lower panel). **(D)** Representative images of tumors in nude mice subcutaneously inoculated with MDA-MB-231 cells with ectopic expression of LOXL4 and control cells treated with saline (control) or BAPN. **(E and F)** Tumor volume (E) and weight (F) in nude mice subcutaneously inoculated with MDA-MB-231 cells with ectopic expression of LOXL4 and control cells treated with saline (control) or BAPN. **(G)** Representative human Ki67 staining pictures of tumor sections (D). Quantification of Ki67 expression (right panel). Scale bar: 30 µm.** (H)** MDA-MB-231 cells stably expressing LOXL4 and control cells were injected into nude mice through the tail vein, and the representative images of bioluminescent images are shown at indicated time points (left panel). Total photon flux at the indicated times is shown in the right panel.** (I)** Representative HE staining pictures of lung sections of control and LOXL4 overexpression group. Arrows indicate metastatic nodules (left panel). Quantification of lung metastatic nodules (right panel).

We also established MDA-MB-231 cells stably expressing LOXL4 and used qRT-PCR and Western blotting to confirm the expression of LOXL4 at both mRNA and protein levels, respectively (Figure 3A). Overexpression of LOXL4 in MDA-MB-231 cells significantly enhanced cell proliferation and migration as determined by colony formation (Figure 3B) and wound healing assays (Figure 3C). To explore whether overexpression of LOXL4 could affect tumorigenesis, MDA-MB-231 cells stably expressing LOXL4 were implanted into nude mice through subcutaneous injection, followed by daily treatment with BAPN (small molecule inhibitor of LOXL4 enzymatic activity) or saline (control). The results showed that mice injected with MDA-MB-231-LOXL4 cells formed larger tumors than those injected with MDA-MB-231-control cells (Figure 3D-G). BAPN treatment resulted in a significantly reduced tumor volume and tumor weight compared to saline treatment (Figure 3D-G), implying the involvement of LOXL4 enzymatic activity in tumorigenesis.

To determine whether overexpression of LOXL4 could affect breast cancer cell metastasis, the MDA-MB-231-control or MDA-MB-231-LOXL4 cells were injected into the tail veins of nude mice. Compared to the control cells, lung metastasis was enhanced in LOXL4-expressing cells, as illustrated by the bioluminescence imaging (Figure 3H). Noticeably, histological examination revealed that mice bearing MDA-MB-231-LOXL4 cells had larger and higher numbers of lung metastasis nodules than mice transplanted with control cells (Figure 3I). Together, these data strongly suggested that LOXL4 had a pivotal role in the promotion of breast cancer tumorigenesis and metastasis *in vitro* and *in vivo*.

### LOXL4 is the direct target of miR-29b and miR-30d regulated by EZH2

**Figure 4:**
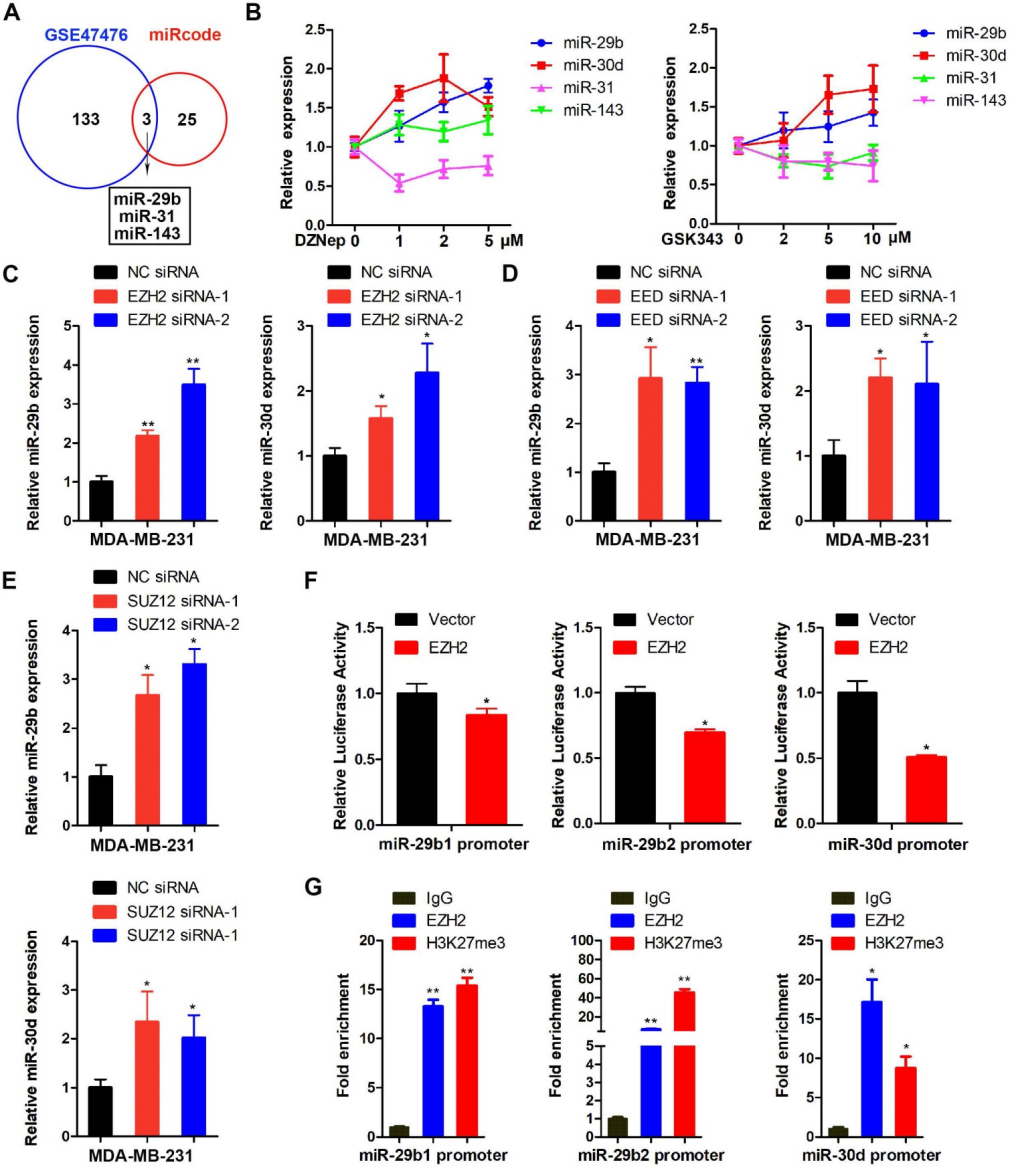
** EZH2 epigenetically suppresses miR-29b or miR-30d expression. (A)** Overlap of miRNAs upregulated in GSE47476 set upon EZH2 inhibition and miRNAs targeting LOXL4 in the miRcode database. **(B)** qRT-PCR of miR-31, miR-143, miR-29b, and miR-30d in MDA-MB-231 cells exposed to different concentrations of DZNep and GSK343. **(C-E)** Knock-down of EZH2, EED, and SUZ12 by siRNAs increased miR-29b and miR-30d gene expression in MDA-MB-231 cells. **(F)** MDA-MB-231 cells co-transfected with a luciferase reporter construct of the miR-29b1 (left panel), miR-29b2 (middle panel) or miR-30d (right panel) promoter (2kb) and pcDNA 3.1-EZH2. Results are expressed as firefly luciferase activity normalized to Renilla luciferase activity. **(G)** ChIP analysis of DNA-chromatin immune-precipitates of MDA-MB-231 cells using anti-EZH2 or anti-H3K27me3 antibody, followed by PCR for amplification of miR-29b1 (left panel), miR-29b2 (middle panel), or miR-30d (right panel) promoter. The corresponding IgG was used as a negative control.

Since the LOXL4 transcription was not directly regulated by EZH2 (Figure S3F), we speculated that miRNAs might serve as a bridge to mediate its transcriptional regulation by EZH2. Therefore, we evaluated the candidate miRNAs that regulated LOXL4 or regulated by EZH2 by interrogating public gene expression databases. Data derived from the GSE47476 set 36 showed that 136 miRNAs were upregulated upon EZH2 inhibition. Another dataset obtained from the miRcode database revealed that LOXL4 might be a potential target of 28 miRNAs. A cross-comparison of the two datasets revealed that 3 miRNAs (miR-31, miR-143, and miR-29b) might be EZH2 targets and regulate LOXL4 expression (Figure 4A). Also, miR-30d could be a candidate miRNA since it was directly regulated by EZH2 epigenetically 36.

Next, we tested the expression of miR-31, miR-143, miR-29b, and miR-30d in MDA-MB-231 cells treated with EZH2 inhibitors DZNep or GSK343. As presented in Figure 4B, miR-29b and miR-30d, but not miR-31 and miR-143, significantly increased following treatment with EZH2 inhibitors compared with the control cells (Figure 4B). Further analysis revealed that the expression of miR-29b and miR-30d was enhanced in EZH2-depleted cells compared with the controls (Figure 4C; Figure S5). Also, knock-down of PRC2 complex subunits, such as SUZ12 and EED, led to increased expression of miR-29b and miR-30d (Figure 4D-E). Promoter activity results showed that EZH2 suppressed the expression of miR-29b and miR-30d at the transcriptional level (Figure 4F). To determine whether EZH2 could bind directly to the promoter regions of miR-29b and miR-30d, we performed ChIP analysis in MDA-MB-231 cells using anti-EZH2 or anti-H3K27me3 antibodies. The results showed that EZH2 specifically bound to the promoter regions of miR-29b and miR-30d (Figure 4G).

**Figure 5:**
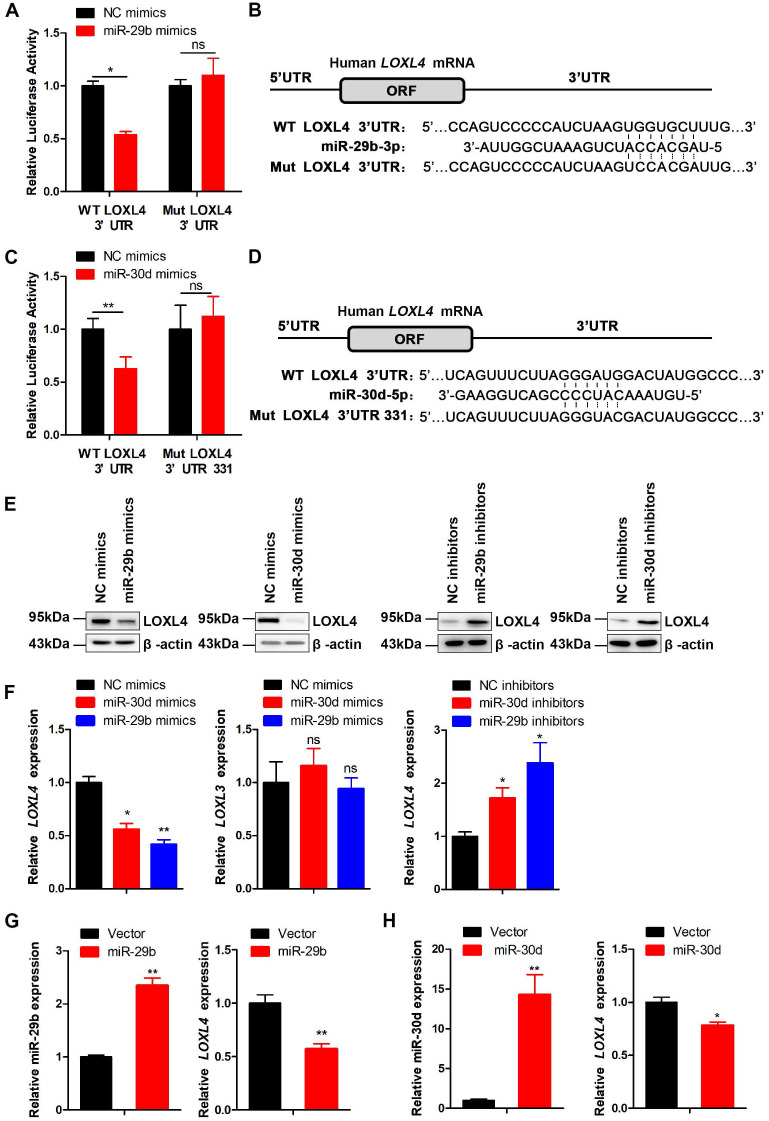
** LOXL4 is the direct target of miR-29b and miR-30d. (A)** WT LOXL4 3'UTR and MUT LOXL4 3'UTR firefly luciferase activities were measured in MDA-MB-231 cells transfected with NC mimics or miR-29b mimics.** (B)** Putative binding site of miR-29b in LOXL4 3'UTR. **(C)** Luciferase reporter assays in MDA-MB-231 cells co-transfected with either WT LOXL4 3'UTR or MUT LOXL4 3'UTR 331 with the predicted miR-30d-binding site mutated and NC mimics or miR-30d mimics. **(D)** Putative binding site of miR-30d in LOXL4 3'UTR.** (E)** Western blotting of LOXL4 in MDA-MB-231 cells transfected with miR-29b/miR-30d mimics or miR-29b/miR-30d inhibitors. **(F)** qRT-PCR of LOXL4 and LOXL3 in MDA-MB-231 cells transfected with miR-29b/miR-30d mimics or miR-29b/miR-30d inhibitors. **(G)** qRT-PCR of miR-29b and LOXL4 in MDA-MB-231 cells stably expressing miR-29b and control cells. **(H)** qRT-PCR of miR-30d and LOXL4 in MDA-MB-231 cells stably expressing miR-30d and control cells.

We further investigated whether LOXL4 was a target of miR-29b and miR-30d by cloning a 527 bp fragment of the LOXL4 3'UTR encompassing the target site downstream of the firefly luciferase gene. The co-transfection of this reporter (WT) with miR-29b or miR-30d caused repression of the luciferase reporter that was specific to LOXL4 binding as the reporter activity was less affected when transfections were repeated with a mutant LOXL4 3'UTR (Figure 5A-D). At the functional level, we predicted that miR-29b or miR-30d binding to the LOXL4 3'UTR would lead to LOXL4 repression. Indeed, ectopic expression of miR-29b or miR-30d mimics caused decreased expression of LOXL4 at both RNA and protein levels (Figure 5E-F). Furthermore, we observed that the expression of LOXL4 at both RNA and protein levels was increased in MDA-MB-231 cells transfected with miR-29b or miR-30d inhibitors (antisense oligonucleotides) (Figure 5E-F). We then established MDA-MB-231 cells that stably expressed miR-29b or miR-30d. As shown in Figure 5G-H, the expression of miR-29b or miR-30d significantly increased, while LOXL4 expression reduced in the stable cell lines, which was consistent with previous results. Collectively, these results implied that LOXL4 was the direct target of miR-29b and miR-30d regulated by EZH2.

### miR-29b and miR-30d inhibit the proliferation and metastasis of breast cancer cells

**Figure 6:**
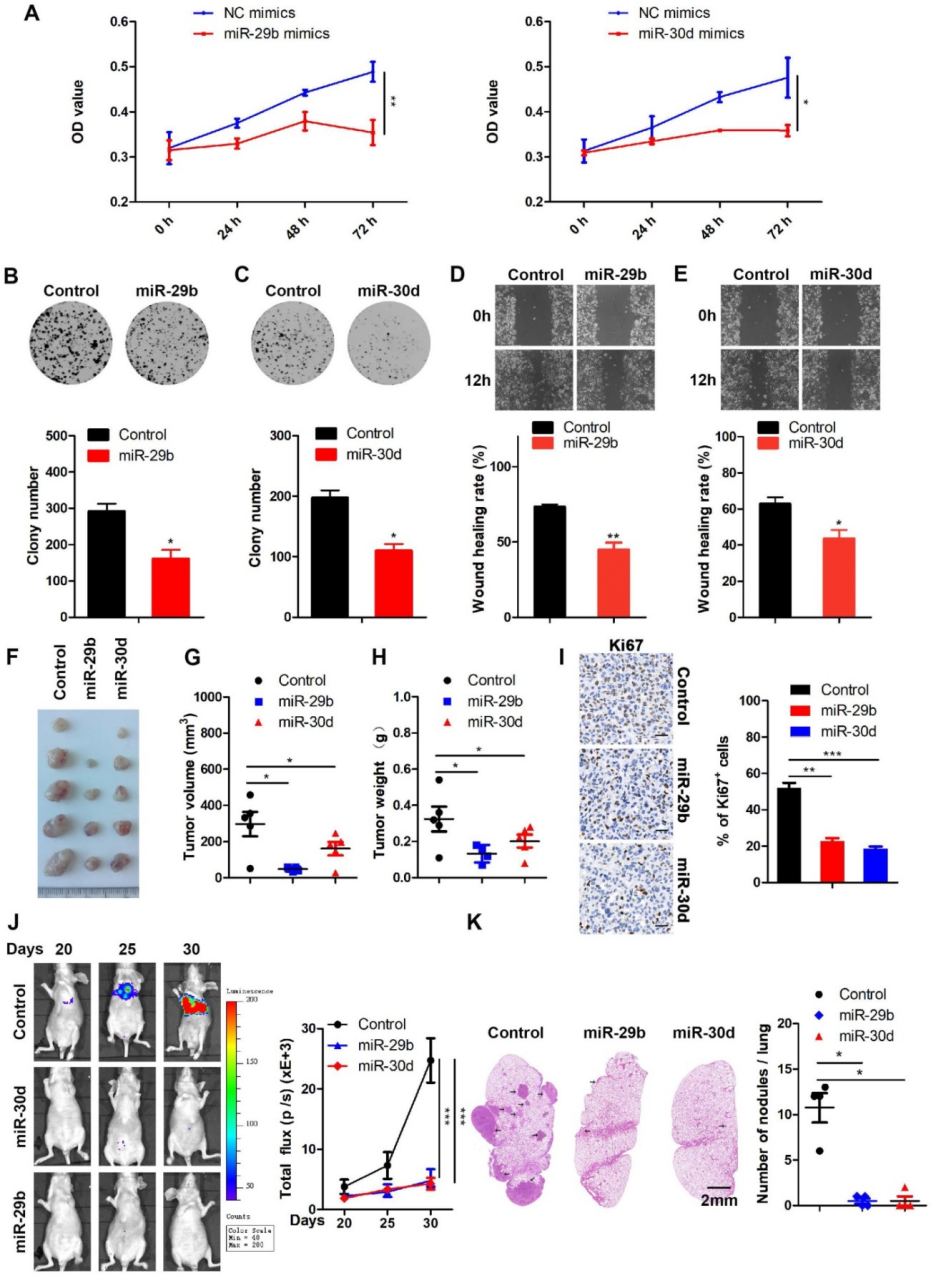
** miR-29b and miR-30d inhibit the proliferation and metastasis of breast cancer cells. (A)** CCK-8 assays to measure cell proliferation at the indicated time points in MDA-MB-231 cells transfected with NC mimics and miR-29b mimics (left panel) or miR-30d mimics (right panel). **(B)** Colony formation assays in MDA-MB-231 cells stably expressing miR-29b and control cells (upper panel). Quantification of colony formation assays (lower panel). **(C)** Colony formation assays in MDA-MB-231 cells stably expressing miR-30d and control cells (upper panel). Quantification of colony formation assays is shown (lower panel). **(D)** Representative images of scratch wound healing assays in MDA-MB-231 cells stably expressing miR-29b and control cells (upper panel). Quantification of wound healing rates is shown (lower panel). **(E)** Representative images of scratch wound healing assays in MDA-MB-231 cells stably expressing miR-30d and control cells (upper panel). Quantification of wound healing rates is shown (lower panel). **(F)** Representative images of tumors in nude mice subcutaneously inoculated with MDA-MB-231 cells ectopic expressing miR-29b, miR-30d, and control cells. **(G and H)** The tumor volume (G) and weight (H) in nude mice subcutaneously inoculated with MDA-MB-231 cells with ectopic expression of miR-29b, miR-30d, and control cells.** (I)** Representative Ki67 staining pictures of tumor sections of control and miR-29b/miR-30d overexpression group (left panel). Quantification of Ki67 expression (right panel). Scale bar: 30 µm. **(J)** MDA-MB-231 cells stably expressing miR-29b or miR-30d and control cells were injected into nude mice through the tail vein, and the representative images of bioluminescent images are shown at indicated time points (left panel). Total photon flux at the indicated times is shown in the right panel. **(K)** Representative HE staining pictures of lung sections of control and miR-29b/miR-30d overexpression group. Arrows indicate metastatic nodules (left panel). Quantification of lung metastatic nodules (right panel).

To investigate the functional roles of miR-29b and miR-30d in breast tumorigenesis, we transfected breast cancer cells with either miR-29b, miR-30d, or control mimics. Following confirmation of miR-29b or miR-30d overexpression by qRT-PCR (Figure S6A), CCK-8 assay was performed to evaluate the effects of increased levels of miR-29b or miR-30d on cell viability. The results showed that miR-29b or miR-30d significantly inhibited the proliferation of breast cancer cells compared to NC mimics (Figure 6A; Figure S6B-C). Cell proliferation and migration in miR-29b or miR-30d-overexpressing and control cells were also determined by colony formation and wound healing assays. As shown in Figure 6B-E and Figure S6D-G, miR-29b or miR-30d overexpression repressed cell proliferation and migration compared with the control cells.

Based on these observations, we next investigated whether overexpression of miR-29b and miR-30d could affect tumorigenesis. When MDA-MB-231 cells stably overexpressing miR-29b or miR-30d were subcutaneously injected into nude mice, smaller tumors were formed compared with control cells (Figure 6F-I).

To explore the effect of miR-29b and miR-30d on tumor metastasis *in vivo*, MDA-MB-231 cells stably expressing miR-29b or miR-30d were injected intravenously into nude mice. As expected, lung metastasis was frequently detected in the control group but rarely in the miR-29b or miR-30d group, indicating that overexpression of these two miRNAs inhibited lung metastasis of breast cancer cells *in vivo* (Figure 6J-K). The analysis of the GSE4589 dataset also showed that the expression of miR-29b or miR-30d was reduced in breast tumors compared to the normal tissues (Figure S7A). These data suggested that miR-29b and miR-30d inhibited the proliferation and metastasis of breast cancer cells.

### EZH2 functions as a positive regulator of LOXL4 through epigenetically repressing miR-29b or miR-30d transcription

As shown in Figure 5A-D, LOXL4 was the direct target of miR-29b and miR-30d, and inhibition of EZH2 reduced LOXL4 expression in breast cancer cells (Figure 1C-E; Figure S2). The EZH2 complex converged at miR-29b and miR-30d promoters to repress their expression (Figure 4C-G). We then asked whether the suppressed expression of LOXL4 induced by EZH2 inhibition was mediated by miR-29b and miR-30d.

**Figure 7:**
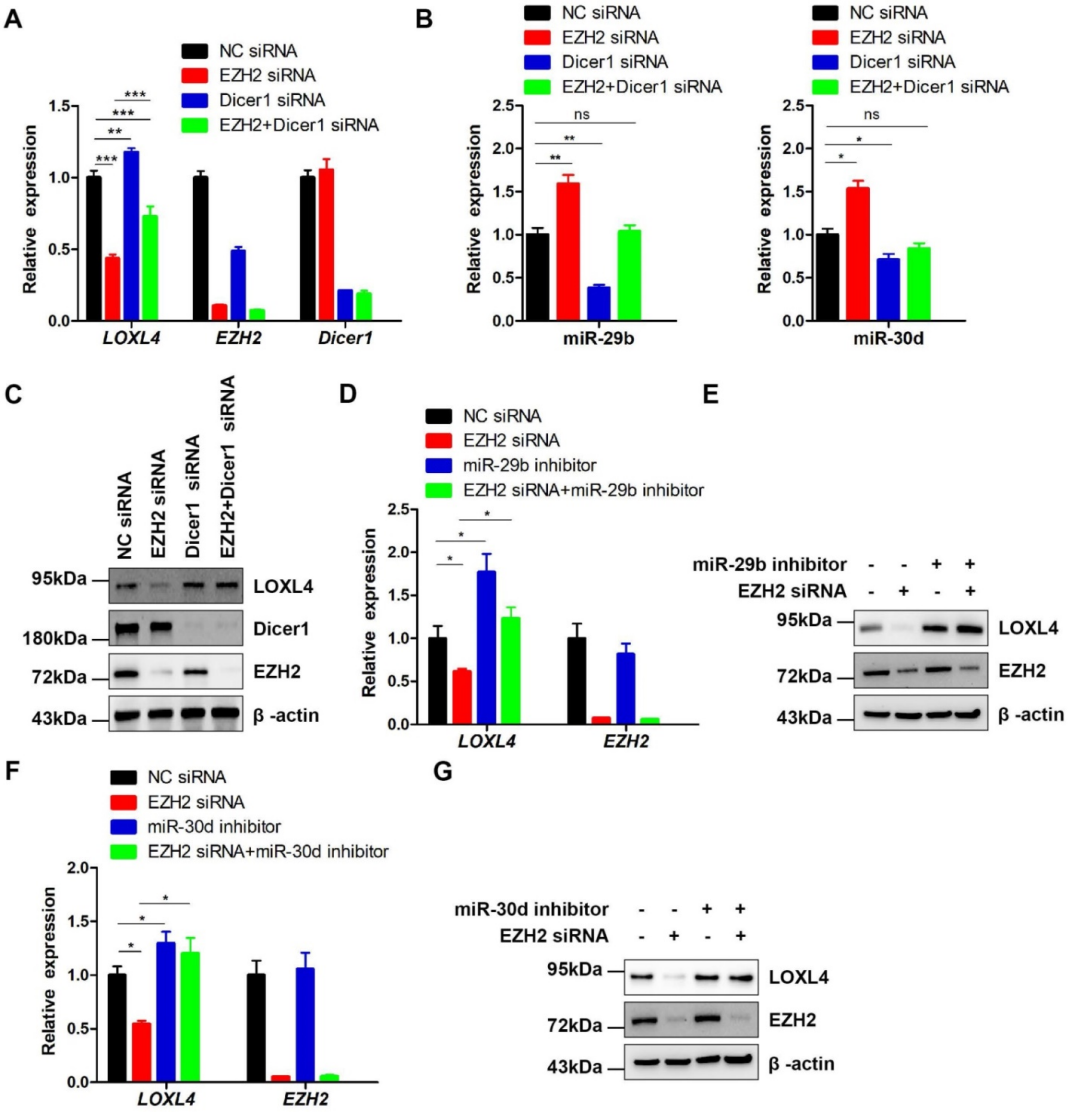
** EZH2 functions as a positive regulator of LOXL4 through epigenetically repressing miR-29b or miR-30d transcription. (A)** qRT-PCR analysis of EZH2, LOXL4, and Dicer1 in MDA-MB-231 cells transfected with NC siRNA, EZH2 siRNA, Dicer1 siRNA, or EZH2 and Dicer1 siRNAs. **(B)** qRT-PCR analysis of miR-29b (left panel) and miR-30d (right panel) in MDA-MB-231 cells transfected with NC siRNA, EZH2 siRNA, Dicer1 siRNA, or EZH2 and Dicer1 siRNAs. **(C)** Immunoblotting analysis of EZH2, LOXL4, and Dicer1 in MDA-MB-231 cells transfected with NC siRNA, EZH2 siRNA, Dicer1 siRNA, or EZH2 and Dicer1 siRNAs. **(D and E)** qRT-PCR (D) and immunoblotting (E) analysis of LOXL4 and EZH2 in MDA-MB-231 cells transfected with NC siRNA, EZH2 siRNA, miR-29b inhibitor, or EZH2 siRNA and miR-29b inhibitor. **(F and G)** qRT-PCR (F) and immunoblotting (G) analysis of LOXL4 and EZH2 in MDA-MB-231 cells transfected with NC siRNA, EZH2 siRNA, miR-30d inhibitor, or EZH2 siRNA and miR-30d inhibitor.

Previous studies have reported that most miRNAs were excised by Dicer from the stems of stem-loop precursors to generate mature miRNA duplexes 37. We, therefore, explored whether co-depletion of EZH2 and Dicer1 could rescue LOXL4 reduction induced by depletion of EZH2 in MDA-MB-231 cells. Indeed, the results demonstrated that co-depletion of EZH2 and Dicer1 rescued LOXL4 reduction induced by depletion of EZH2 in MDA-MB-231 cells at both mRNA and protein levels. The increased expression of miR-29b and miR-30d upon EZH2 depletion was also inhibited by the addition of EZH2 and Dicer1 siRNAs in MDA-MB-231 cells (Figure 7A-C).

To investigate whether miR-29b and miR-30d were critical regulators for reduced expression of LOXL4 induced by EZH2 knock-down, we performed a rescue experiment to test whether miR-29b and miR-30d inhibition would increase LOXL4 expression in EZH2 knock-down cells. The results showed that miR-29b and miR-30d inhibitors rescued the reduced expression of LOXL4 at both mRNA and protein levels upon EZH2 knock-down (Figure 7D-G). Hence, our results demonstrated that miR-29b and miR-30d acted as key mediators in EZH2-mediated regulation of LOXL4.

### The EZH2-miR-29b/miR-30d-LOXL4 signaling pathway regulates macrophage infiltration and collagen remodeling in breast cancer

**Figure 8:**
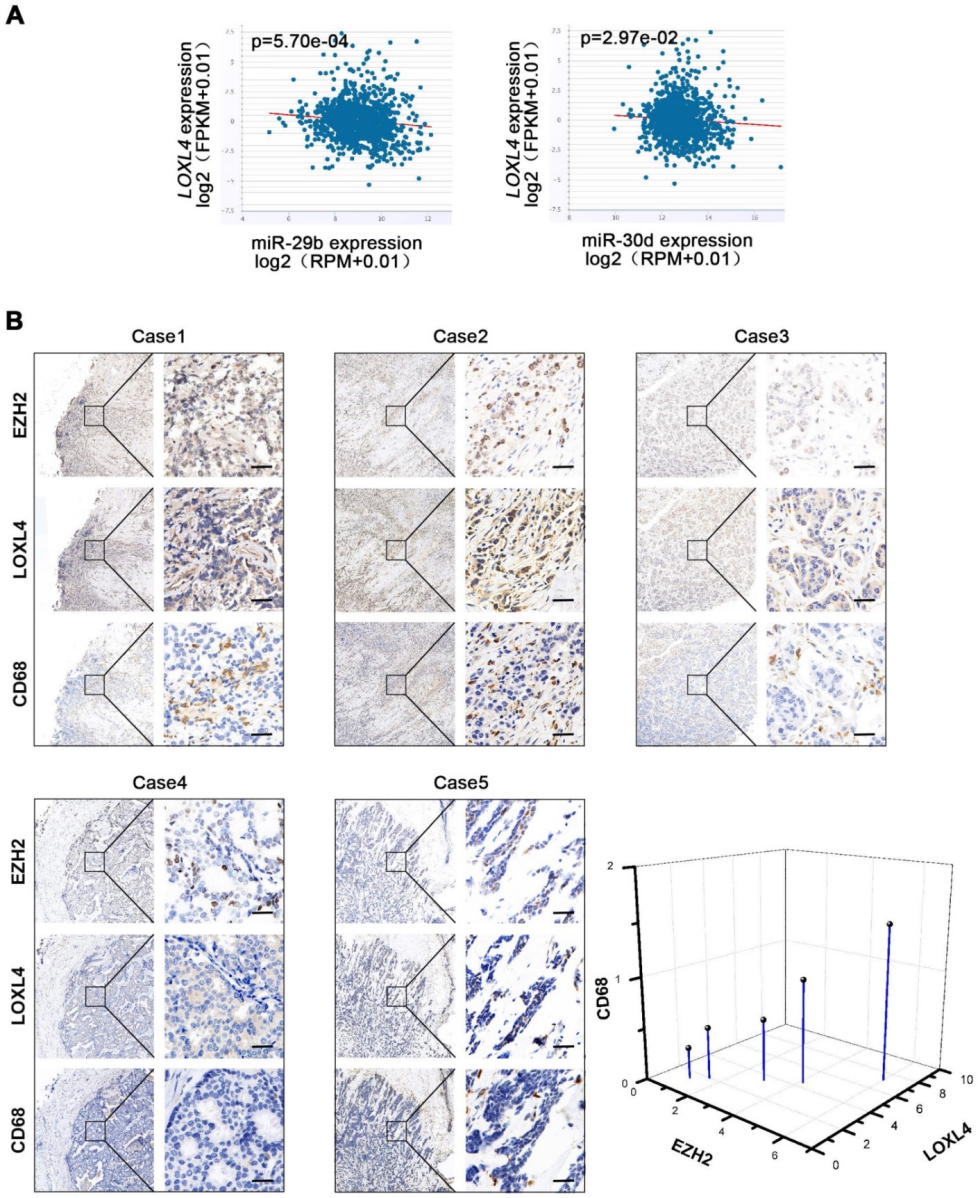
**Correlation analysis of EZH2, miR29b/miR-30d, LOXL4, and CD68. (A)** Correlation analysis of EZH2 and miR29b/miR-30d in invasive breast carcinoma using starBase v2.0 (http://starbase.sysu.edu.cn/). **(B)** Representative photographs of the EZH2, LOXL4, and CD68 staining in breast cancer patient tissues. Quantification of the normalized staining intensity is shown. Scale bar: 30 µm.

We evaluated the correlation between EZH2 and miR-29b/30d in breast cancer cells and found an inverse correlation between miR-29b/30d and LOXL4 mRNA by starBase v2.0 analysis (Figure 8A). A positive correlation between EZH2 and LOXL4 expression was also detected in breast cancer patient samples (Figure 8B). Given that EZH2-miR-29b/miR-30d-LOXL4 signaling pathway was associated with tumor progression, we next asked whether the expression of these genes was associated with poor prognosis in breast cancer patients. Indeed, a correlation between high EZH2 expression and shorter overall survival was observed in patients with estrogen receptor (ER)-positive breast cancers, while low expression of LOXL4 and high level of miR-30d was associated with good prognosis (Figure S7B-C). Furthermore, a low level of miR-29b was associated with faster disease progression and poor prognosis of triple-negative breast cancer (TNBC) subtype (Figure S7C). However, due to the low number of patients in breast cancer subtypes, no other significant correlation was observed.

**Figure 9:**
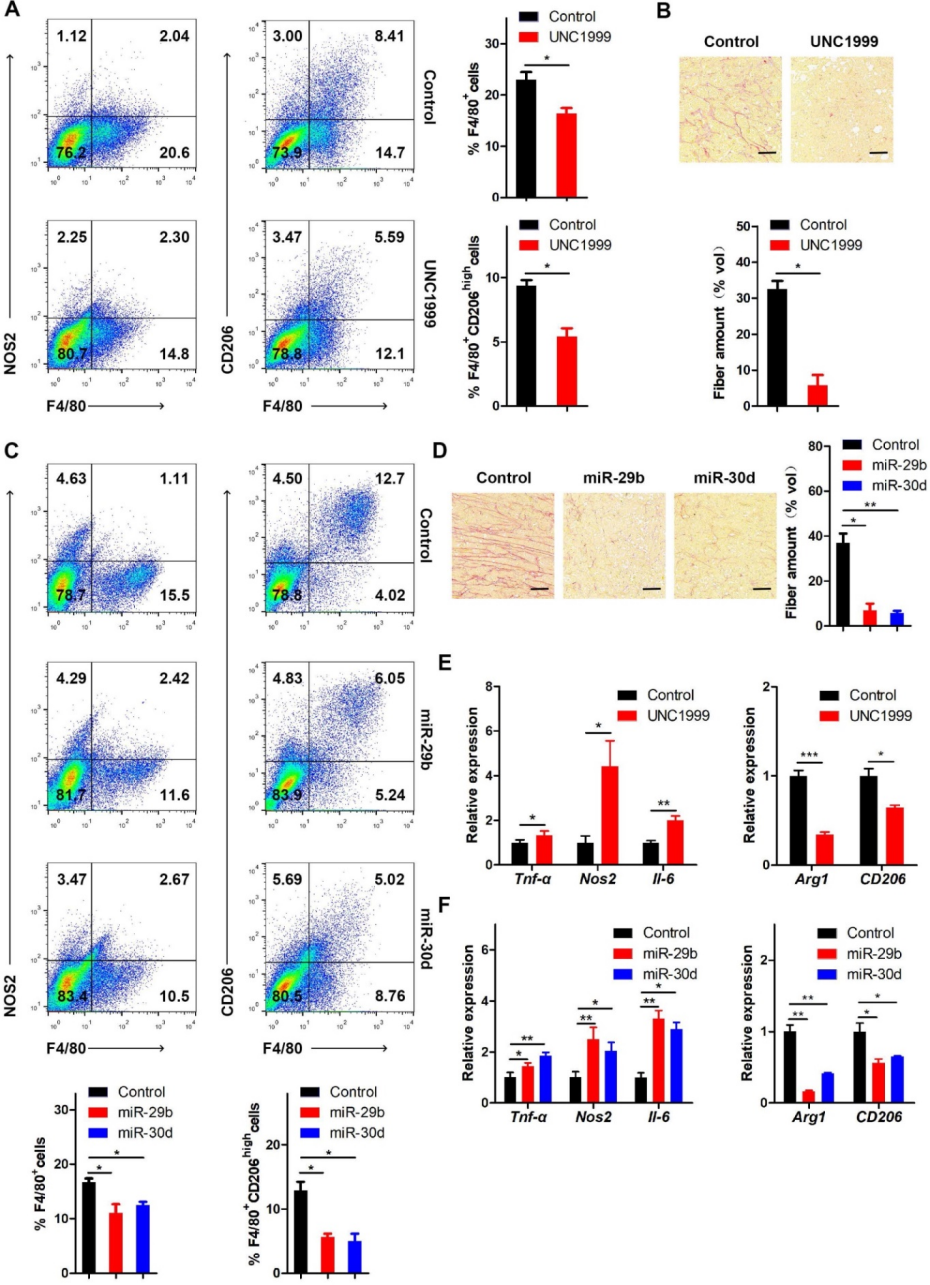
**The EZH2-miR-29b/miR-30d-LOXL4 signaling pathway regulates macrophage infiltration and collagen remodeling in breast cancer. (A)** Flow cytometry analysis of macrophage subpopulations of the 4T1 tumors in mice injected intraperitoneally with 4T1 cells treated with UNC1999. The percentages of (F4/80^+^) TAMs and (F4/80^+^CD206^high^) M2-like TAMs were calculated.** (B)** Representative images of collagen fibrils from the 4T1 tumors in mice injected intraperitoneally with 4T1 cells treated with UNC1999.** (C)** Flow cytometry analysis of macrophage subpopulations of the 4T1 tumors in mice injected intraperitoneally with 4T1 cells overexpressing miR-29b or miR-30d. The percentages of (F4/80^+^) TAMs and (F4/80^+^CD206^high^) M2-like TAMs were calculated.** (D)** Representative images of collagen fibrils from the 4T1 tumors in mice injected intraperitoneally with 4T1 cells overexpressing miR-29b or miR-30d.** (E)** F4/80^+^ TAMs were isolated from 4T1 tumors of mice injected intraperitoneally with 4T1 cells treated with UNC1999. M1 and M2 macrophage-associated gene expression were assessed by qRT-PCR.** (F)** F4/80^+^ TAMs were isolated from 4T1 tumors of mice injected intraperitoneally with 4T1 cells overexpressing miR-29b or miR-30d. M1 and M2 macrophage-associated gene expression was assessed by qRT-PCR. Scale bar: 30 µm.

It has been reported that LOX functions as a potent macrophage chemoattractant 38. Therefore, we used CD68 staining to examine macrophage infiltration in human breast cancer tissues. The results showed that macrophage infiltration was positively correlated with EZH2 and LOXL4 expression (Figure 8B), implying that tumor-associated macrophages (TAM) may play a vital role in EZH2/LOXL4-mediated progression of breast cancer. Because of this observation, we investigated whether EZH2 and miR-29b/miR-30d were involved in inducing macrophage infiltration *in vivo.* The results showed that inhibition of EZH2 by UNC1999 reduced the percentages of TAMs (F4/80^+^) and M2-like TAMs (F4/80^+^CD206^high^) (Figure 9A) as well as collagen remodeling in 4T1 tumors (Figure 9B). Consistent with these observations, 4T1 tumors with miR-29b/miR-30d overexpression exhibited decreased percentages of TAMs (F4/80^+^) and M2-like TAMs (F4/80^+^CD206^high^) and reduced collagen fiber content (Figure 9C-D).

Next, we isolated F4/80-positive TAMs and analyzed the expression of M1 and M2 associated genes by qRT-PCR. As displayed in Figure 9E, the expression of M2 associated genes (Arg1 and CD206) was attenuated while that of M1 associated genes (Tnf-α, Nos2, and IL-6) was increased in TAMs from UNC1999-treated 4T1 tumors compared with control tumors. Similar results were obtained in 4T1 tumors with overexpression of miR-29b/miR-30d compared to control tumors (Figure 9F). Thus, our data indicated that the EZH2-miR-29b/miR-30d-LOXL4 signaling axis regulated macrophage activation and collagen remodeling in breast cancer.

## Discussion

Our study showed that the EZH2-miR-29b/miR-30d-LOXL4 axis was involved in the progression of breast cancer cells. Specifically, we demonstrated that EZH2 promoted LOXL4 expression through epigenetically repressing the expression of miR-29b and miR-30d. The dual-luciferase gene reporter assay showed that LOXL4 was a direct target of miR-29b and miR-30d. Overexpression of miR-29b and miR-30d inhibited LOXL4 expression, resulting in impaired proliferation, migration, tumorigenesis, and metastasis of breast cancer cells.

As a crucial epigenetic regulator of gene expression, EZH2 is associated with multiple aspects of many carcinomas, such as tumor initiation, invasiveness, metastasis, angiogenesis, and chemoresistance 10. EZH2 has been shown to function as a major oncogene involved in proliferation, migration, and metastasis of breast cancer cells 39-41, but the mechanisms are poorly understood. As the ECM is a highly dynamic and complex molecular network present in all tissues, its components are thought to be significant biological players in breast cancer progression and metastasis 16, 17. To date, how EZH2 regulates the ECM in the progression of breast cancer remains unknown. In the current study, we demonstrated that blocking EZH2 activity by EZH2 inhibitors or EZH2 siRNAs significantly reduced the expression of LOXL4 associated with low H3K27me3 expression, suggesting that coordinated epigenetic silencing may exist in breast cancer cells (Figure 1C-E; Figures S1A and S2). So far, scant information is available on the role of LOXL4 in human malignancies, and there are conflicting conclusions regarding the effects of LOXL4 in cancers 28-32. Herein, we found that LOXL4 was significantly correlated with breast cell proliferation and migration *in vitro* and tumor metastasis *in vivo.* We performed gain- and loss-of-function studies, and provided strong evidence that LOXL4 was a key determinant of breast cancer tumorigenesis and metastasis (Figures 2D-J and 3D-I), in contrast to a previous study showing that LOXL4 was downregulated in breast cancer tissues 42.

Relevant to our study, as one of the five LOX family members, LOX significantly promoted macrophage infiltration and tumor progression in glioblastoma multiforme 38. In our study, we observed that LOXL4 expression was positively correlated with EZH2 expression and macrophage infiltration, evidenced by CD68 staining in human breast cancer tissues (Figure 8B). EZH2 inhibition or miR-29b/miR-30d overexpression reduced the percentages of TAM (F4/80^+^) and M2-like TAMs (F4/80^+^CD206^high^) in 4T1 tumors (Figure 9A and C). These results indicated that EZH2-miR-29b/miR-30d-LOXL4 signaling pathway regulated macrophages infiltration and activation in the progression of breast cancer.

miRNAs and their targets constitute an orchestrated signaling network in breast cancer. The miRNA expression analysis has revealed several novel and promising markers for diagnosis, prognosis, and treatment of breast cancer 43, 44. Shinden et al. have shown that miR-29b is a prognostic marker in breast cancer patients 45. Similarly, Liu et al. have reported that down-regulation of miR-29b in carcinoma-associated fibroblasts facilitates cell growth and metastasis of breast cancer 46. Thus, the high expression of miR-29b is closely associated with a good prognosis in breast cancer. Also, miR-30 family members play various roles in tumorigenesis and suppression, but they mainly serve as tumor suppressors in breast cancer progression and metastasis 47-49.

Recently, the epigenetic regulation of tumor-suppressive microRNAs by EZH2 has been shown to be critical in tumorigenesis 50, 51. It has been reported that EZH2 directly inhibits miR-29, miR-26, miR-101, and miR-30 families in B cell lymphomas, chronic lymphocytic leukemia, hepatocellular carcinoma, malignant peripheral nerve sheath tumor cells, and prostate cancer 33, 36, 52-54. However, the potential role of EZH2 in driving breast cancer progression still needed further characterization.

In our study, we found that EZH2 knockdown-mediated inhibition of cell proliferation depended on the repression of LOXL4 and upregulation of miR-29b and miR-30d. Our data provide valuable insights into the EZH2 role as a key epigenetic regulator of the ECM and also highlight the importance of performing preclinical studies in breast cancer that target EZH2 and LOXL4. The understanding of molecular mechanisms underlying the EZH2-miR-29b/miR-30d-LOXL4 axis affords a novel insight in diagnosis, prognosis, and molecularly targeted therapy of breast cancer and may provide a novel therapeutic approach for breast cancer treatment in the clinic. Combined assessment of EZH2, LOXL4, miR-29/miR-30d and macrophage infiltration may be a more effective diagnostic method for breast cancer.

## Conclusions

Our study has illustrated that EZH2-miR-29b/miR-30d-LOXL4 signaling pathway is critical for the proliferation and metastasis of breast cancer cells. To our knowledge, we have shown, for the first time that LOXL4 is regulated by EZH2 through epigenetic repression of miRNAs. Our work also suggests that epigenetic modulation represents a potential therapeutic target for breast cancer to restrain macrophage activation. These results may open up potential novel avenues for exploring efficient therapeutic strategies for breast cancer treatment.

## Supplementary Material

Supplementary figures and tables.

## Abbreviations

EZH2: Enhancer of Zeste Homolog 2
ECM: Extracellular matrix
LOXL4: Lysyl oxidase-Like 4
HE: Haematoxylin and eosin
IHC: Immunohistochemistry
CCK-8: Cell Counting Kit-8
ChIP: Chromatin immunoprecipitation
qRT-PCR: Reverse transcription quantitative real-time PCR
shRNA: Short hairpin RNA
TAM: Tumor-associated macrophages
