# Supplementary material for: EZH2-mediated Epigenetic Silencing of miR-29/miR-30 targets LOXL4 and contributes to Tumorigenesis, Metastasis, and Immune Microenvironment Remodeling in Breast Cancer
Source: Theranostics. 2020 Jul 9;10(19):8494–512. doi: 10.7150/thno.44849 (PMC7392008; doi:10.7150/thno.44849)
Supplement: Supplementary file 1 — Supplementary figures and tables. [file thnov10p8494s1.pdf]

EZH2-mediated epigenetic silencing of miR-29/miR-30 targets LOXL4 and contributes to tumorigenesis, metastasis and immune microenvironment remodeling in breast cancer

Huilong Yin [*], Yidi Wang [*], Ye Wu [*], Xiang Zhang, Xiaofang Zhang, Jun Liu, Ting Wang, Jing Fan, Jianyong Sun, Angang Yang [†] and Rui Zhang [†]

[†] Correspondence should be sent to: ruizhang@fmmu.edu.cn

This file includes:

Supplementary Text
Supplementary Figures 1 to 7
Supplementary Tables 1 to 4

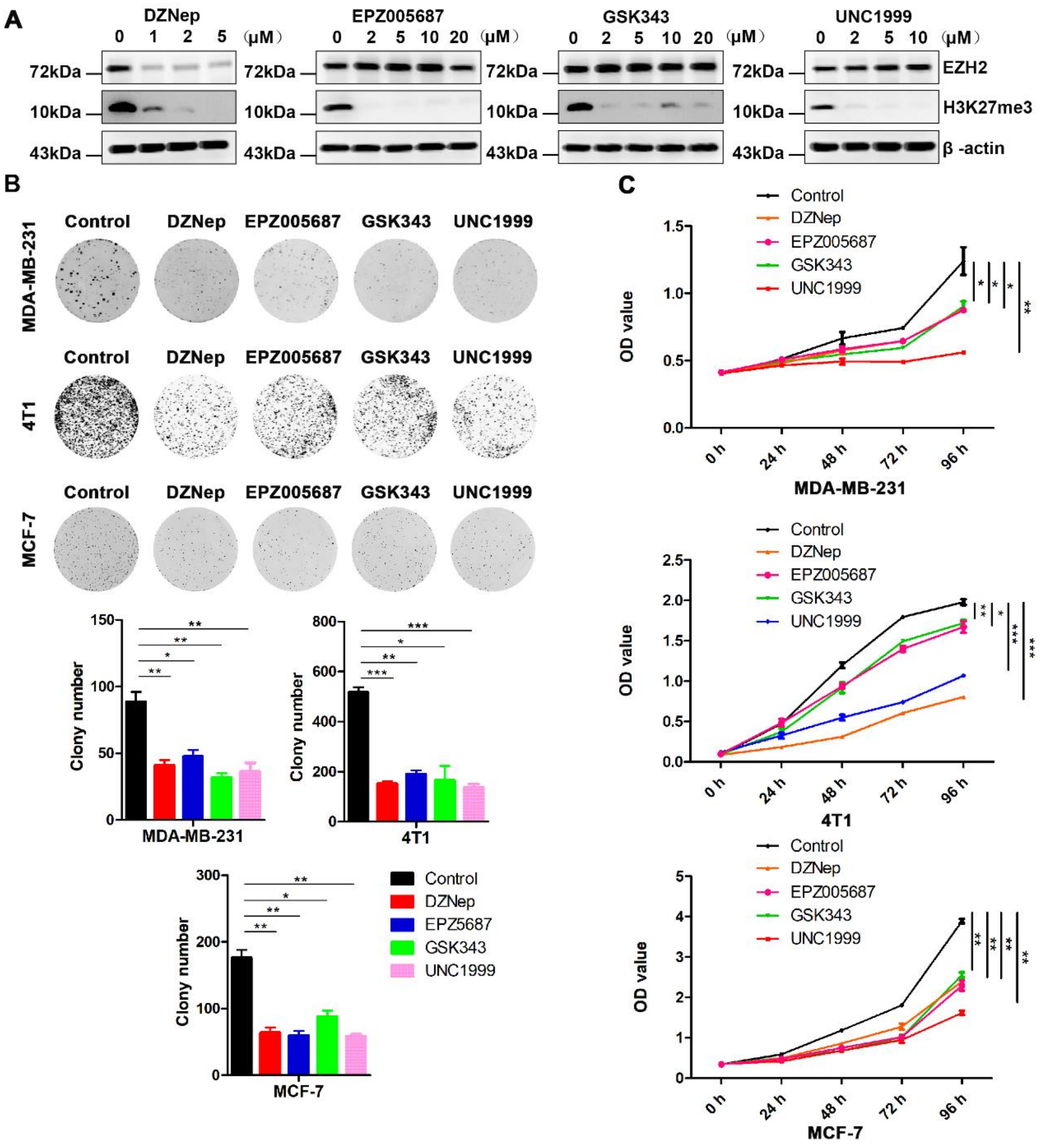

Supplementary Figure 1. EZH2 inhibitors block cell proliferation. (A) MDA-MB-231 cells were treated with different concentrations of DZNep, GSK343, UNC1999, or EPZ005687 for 48 h. H3K27me3 expression was detected by immunoblotting. **(B)** Colony formation assays of breast cancer cells in the presence of DMSO, DZNep, EPZ005687, GSK343, or UNC1999 on day 14 (upper panel). Quantification of colony formation assays (lower panel). **(C)** Breast cancer cells were treated with DMSO, DZNep, EPZ005687, GSK343, or UNC1999, and the cell viability was determined

using the CCK-8 assay.

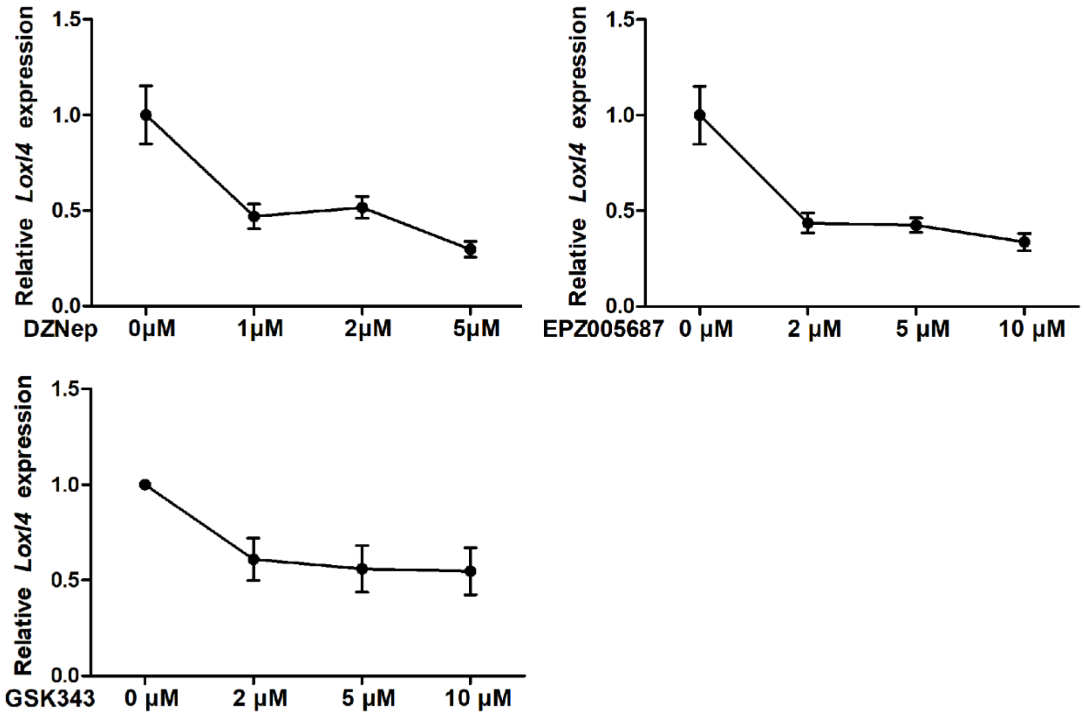

Supplementary Figure 2. EZH2 inhibitors decrease LOXL4 expression. qRT-PCR analysis of LOXL4 in 4T1 cells treated with different concentrations of DZNep, EPZ005687, or GSK343.

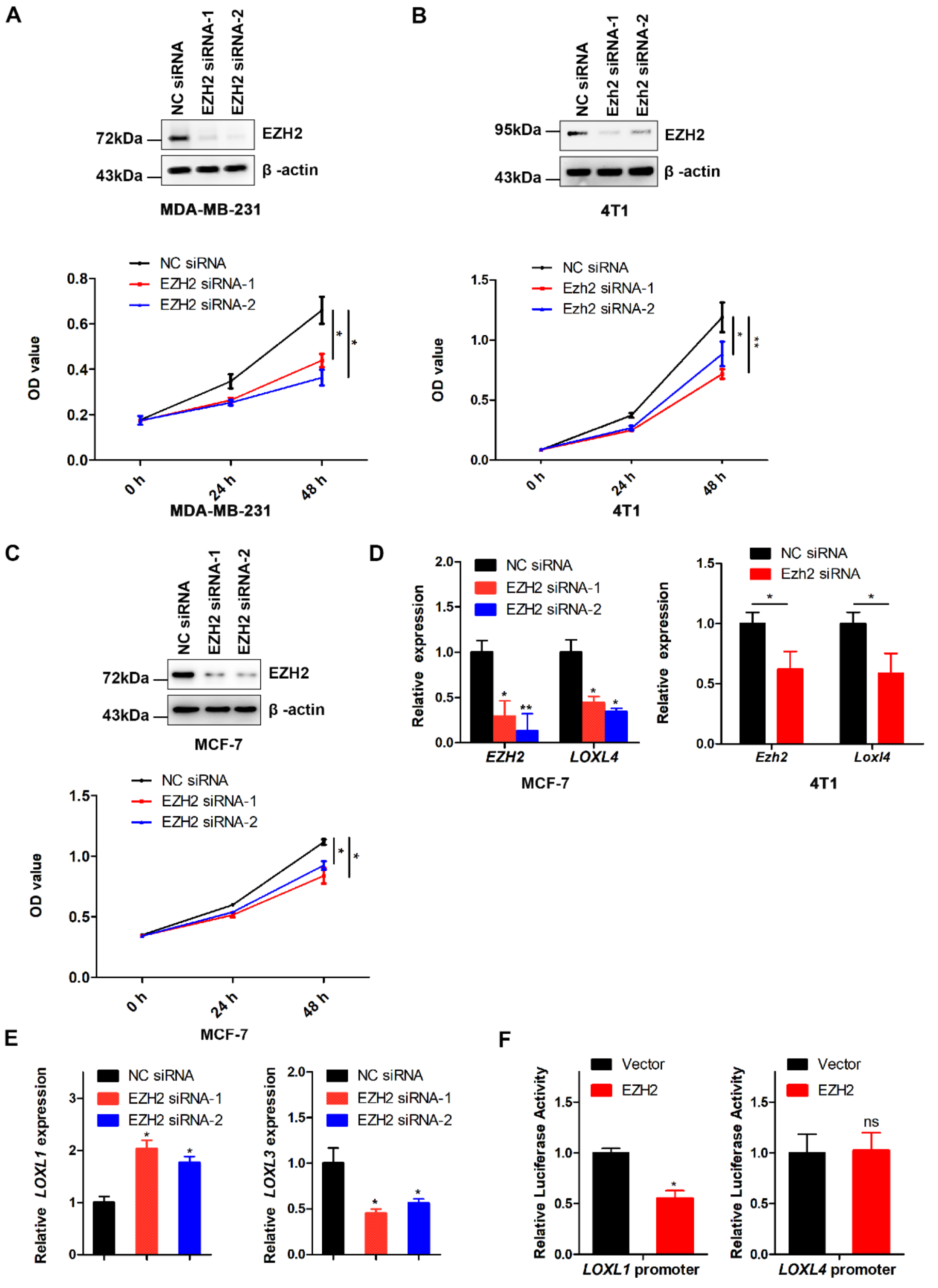

Supplementary Figure 3. Knock-down of EZH2 blocks cell proliferation and decreases the expression of LOXL4. (A-C) MDA-MB-231 (A), 4T1 （B）and MCF-7 （C） cells were transfected with NC siRNA or EZH2 siRNA. Efficiency of EZH2 siRNAs was examined by Western blotting (upper panel). Cell viability was determined

by the CCK-8 assay (lower panel). **(D)** MCF-7 and 4T1 cells were transfected with NC siRNA or EZH2 siRNA. Cells were harvested 48 h after transfection for analysis of EZH2 and LOXL4 mRNA expression by qRT-PCR. **(E)** qRT-PCR analysis of LOXL1 and LOXL3 in MDA-MB-231 cells transfected with NC siRNA or EZH2 siRNA. **(F)** Relative luciferase activity was conducted after cells were transfected with LOXL1 or LOXL4 promoter (2kb) and pcDNA 3.1-EZH2.

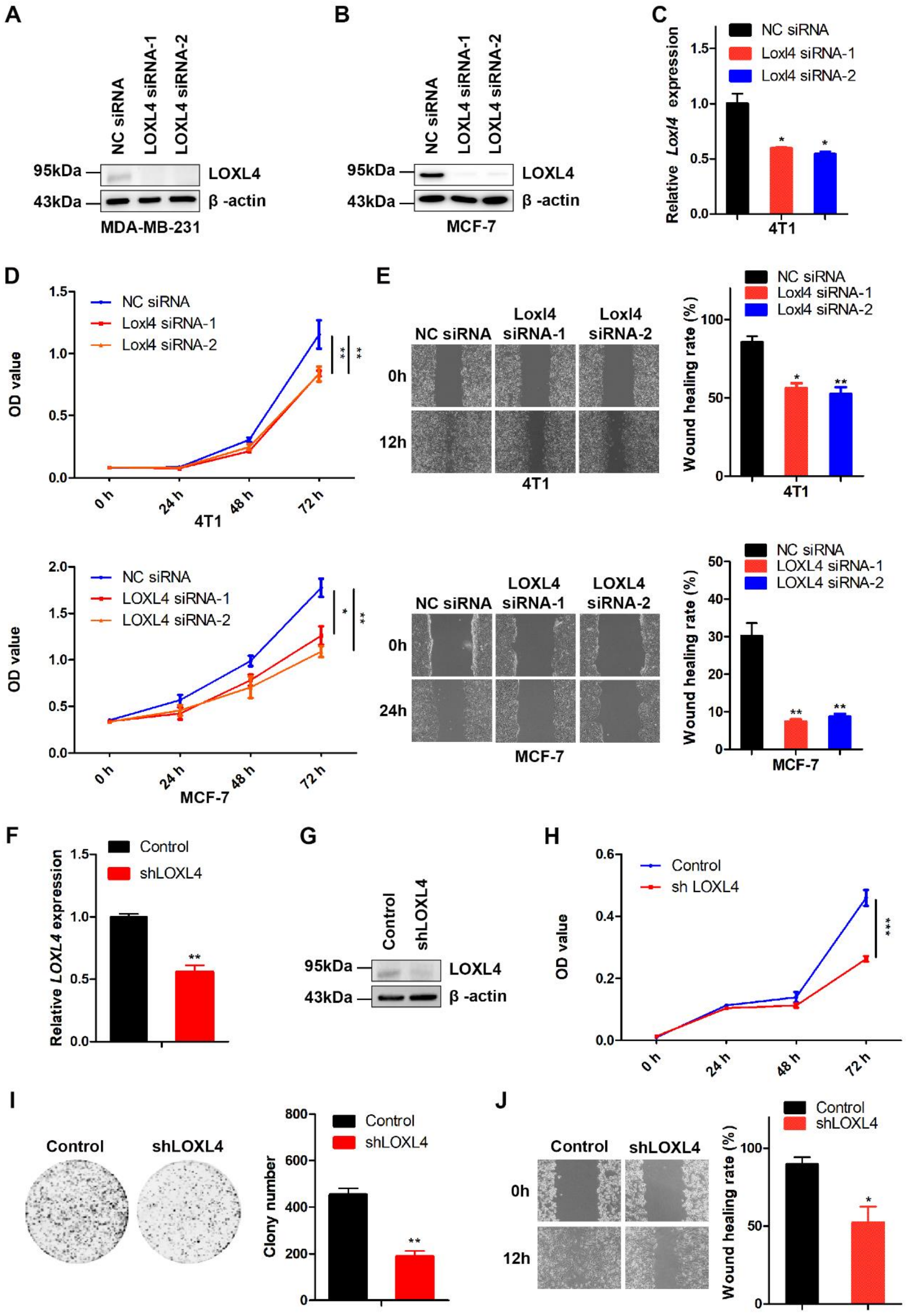

Supplementary Figure 4. LOXL4 knockdown inhibits cell proliferation and migration *in vitro*. (A and B) Western blotting analysis of LOXL4 in MDA-MB-231 and MCF-7 cells transfected with NC siRNA or LOXL4 siRNA. **(C)** qRT-PCR analysis

of Loxl4 in 4T1 cells transfected with NC siRNA or Loxl4 siRNA. **(D)** Cell proliferation analysis was performed in 4T1 and MCF-7 cells transfected with NC siRNA or LOXL4 siRNA. **(E)** Representative images of scratch wound healing assays in 4T1 and MCF-7 cells transfected with NC siRNA or LOXL4 siRNA (left panel). Quantification of wound healing rates (right panel). **(F and G)** qRT-PCR (F) and Western blotting (G) analysis of LOXL4 in MDA-MB-231 cells with LOXL4 stable knockdown and control cells. **(H)** Cell proliferation analysis was performed in MDA-MB-231 cells with stable expression of control shRNA or LOXL4 shRNA. **(I)** Colony formation assays in MDA-MB-231 cells with LOXL4 stable knockdown and control cells. (left panel). Quantification of colony formation assays is shown (right panel). **(J)** Representative images of scratch wound healing assays in MDA-MB-231 cells with LOXL4 stable knock down and control cells (left panel). Quantification of wound healing rates (right panel).

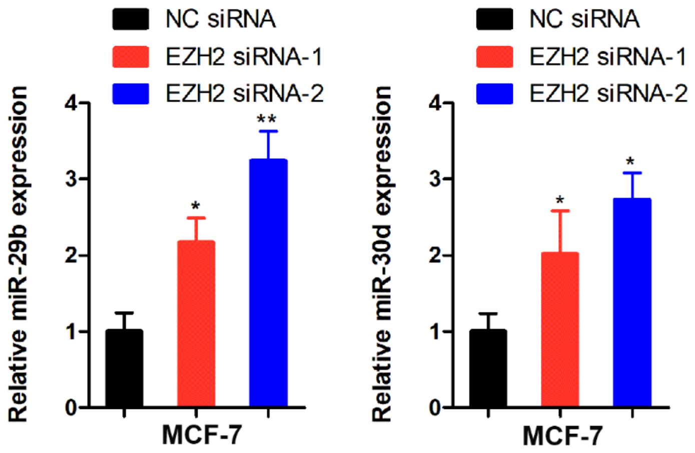

Supplementary Figure 5. Increased expression of miR-29b and miR-30d in EZH2-depleted cells. Knock-down of EZH by siRNAs increased miR-29b and miR-30d gene expression in MCF-7 cells.

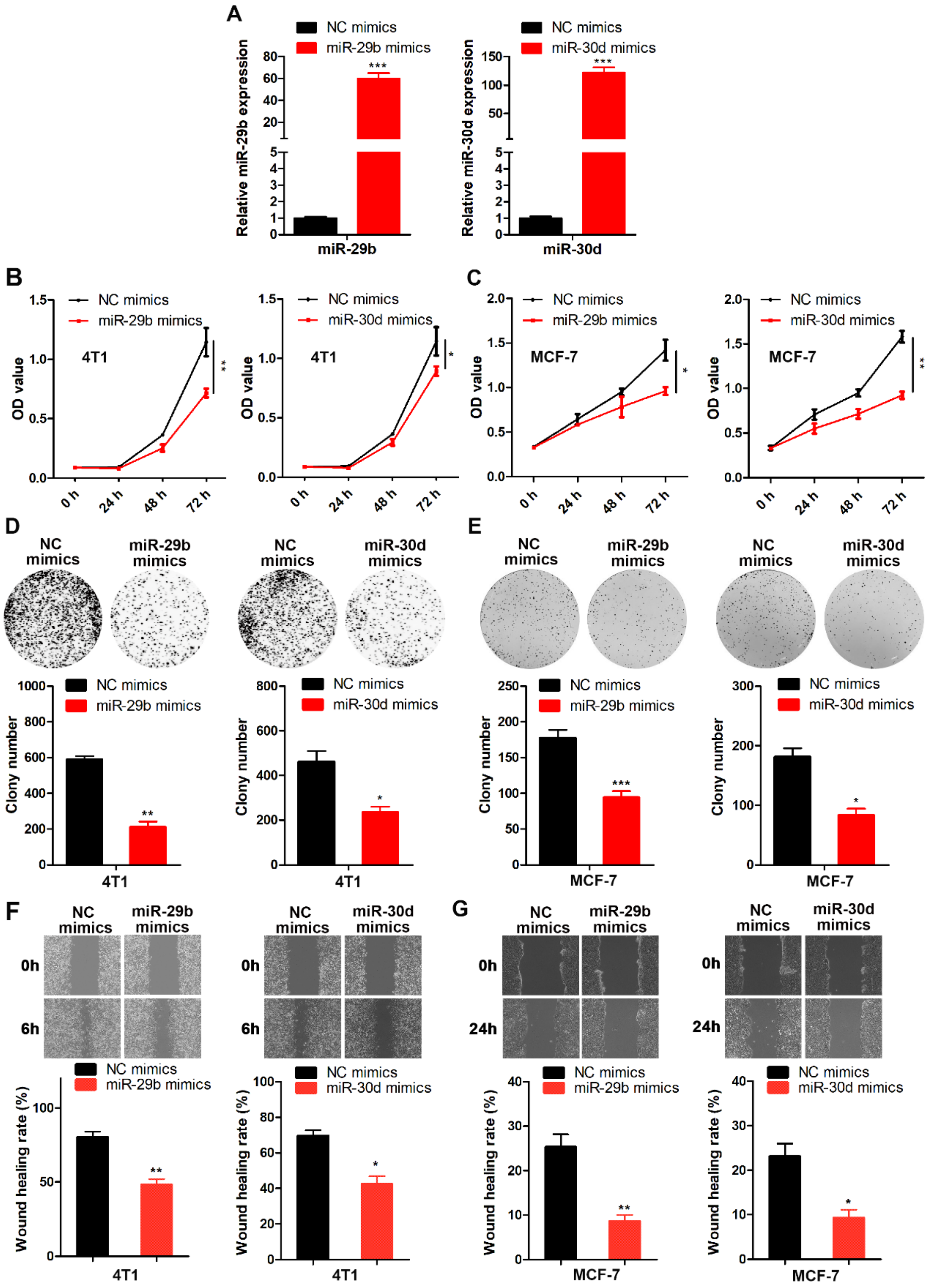

Supplementary Figure 6. miR-29b and miR-30d inhibit the proliferation and migration in breast cancer cells. (A) qRT-PCR analysis of miR-29b (left panel) and miR-30d (right panel) in MDA-MB-231 cells transfected with NC mimics, miR-29b mimics or miR-30d mimics. **(B and C)** CCK-8 assay was used to measure cell

proliferation at the indicated time points in 4T1 (B) and MCF-7 (C) cells transfected with NC mimics and miR-29b mimics or miR-30d mimics. **(D and E)** Colony formation assay in 4T1 (D) and MCF-7 (E) cells transfected with NC mimics and miR-29b mimics or miR-30d mimics (upper panel). Quantification of colony formation assays (lower panel). **(F and G)** Representative images of scratch wound healing assays in 4T1 (F) and MCF-7 (G) cells transfected with NC mimics and miR-29b mimics or miR-30d mimics (upper panel). Quantification of wound healing rates is shown (lower panel).

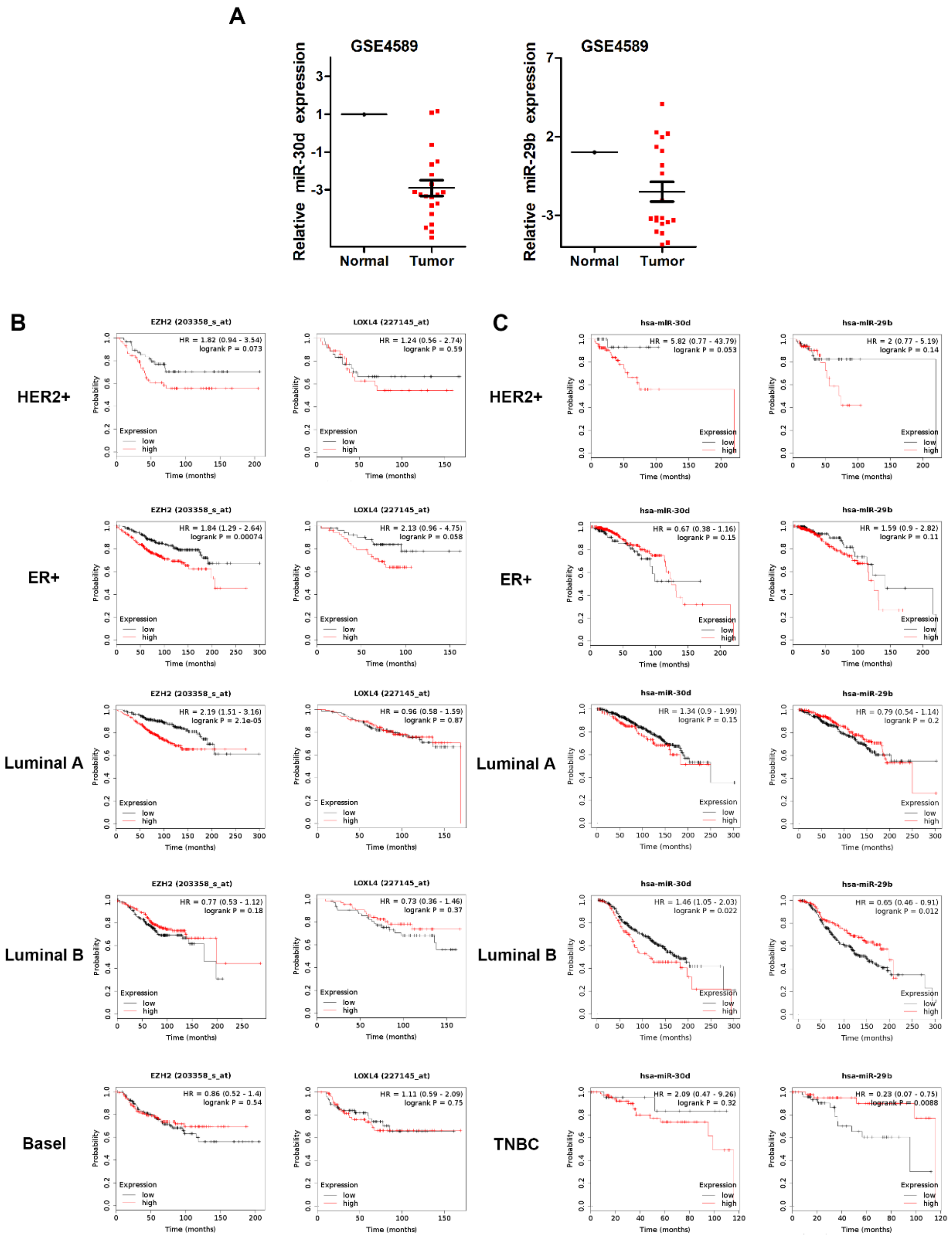

Supplementary Figure 7. EZH2-miR-29b/miR-30d-LOXL4 signaling pathway correlates with poor prognosis in human cancers. (A) mRNA expression levels of miR-29b and miR-30d in breast cancer tissues compared with the normal liver tissues using the GSE4589 dataset. **(B)** Probability of overall survival for breast cancer patients

with HER2-positive (HER2+), ER-positive (ER+), luminal A, luminal B or basal breast cancer subtypes according to EZH2 and LOXL4 gene expression levels using Breast Km Plotter online survival analysis. **(C)** Comparison of overall survival of breast cancer patients with different miR-29b and miR-30d expressions with the Breast Km Plotter online tool.

Supplementary Table 1. Clinicopathological characteristics of BRCA patients analyzed

Case#	TNM stage	Type	ER	PR	Her	Clinical classification	Grade
		0=DCIS<br>1=IDC<br>2=ILC<br>3=DCIS+I<br>4=OTHER	0=N<br>1=1	0=N<br>1=1	0=-<br>1=1+		
1	T2N0Mx	1	-	-	0	TN	IA
2	T2N0M0	2	3	3	2	Luminal B	IA
3	T1cN0M0	1	2	1	3	Luminal B2	IA
4	T2N0M0	1	3	2	0	Luminal B1	IIA
5	T1cN0M0	1	3	2	1	Luminal A	IIIA

Supplementary Table 2. siRNA sequences

	sense（5'-3'）	antisense（5'-3'）
Ezh2-siRNA-1（Mus musculus ）	GAAAGAUCUAGAGGAUAAUTT	AUUAUCCUCUAGAUCUUUCTT
Ezh2-siRNA-2（Mus musculus ）	CUCGGUGUCAAACACCAAUTT	AUUGGUGUUUGACACCGAGTT
Loxl4-siRNA-1（Mus musculus ）	CCAGUCAGACAUCUGUCAATT	UUGACAGAUGUCUGACUGGTT
Loxl4-siRNA-2（Mus musculus ）	GCUAUGCAUGUGCCAACUUTT	AAGUUGGCACAUGCAUAGCTT
EZH2-siRNA-1（Homo sapiens ）	GACUCUGAAUGCAGUUGCUTT	AGCAACUGCAUUCAGAGUCUU
EZH2-siRNA-2（Homo sapiens ）	GGAUGGUACUUUCAUUGAATT	UUCAAUGAAAGUACCAUCCTT
SUZ12-siRNA-1（Homo sapiens ）	GUCGCAACGGACCAGUUAATT	UUAACUGGUCCGUUGCGACTT
SUZ12-siRNA-2（Homo sapiens ）	GACUACAGAUCUACAAACATT	UGUUUGUAGAUCUGUAGUCTT
EED-siRNA-1（Homo sapiens ）	AAGCACUAUGUUGGCCAUGGATT	UCCAUGGCCAACAUAGUGCUUTT
EED-siRNA-2（Homo sapiens ）	UGGUGCUGCUAUUCGACAATT	UUGUCGAAUAGCAGCACCATT
LOXL4-siRNA-1（Homo sapiens ）	GCUGAAGAGCCUGACGAAUTT	AUUCGUCAGGCUCUUCAGCTT
LOXL4-siRNA-2（Homo sapiens ）	CCAAGUCUGCGGAUCACAUTT	AUGUGAUCCGCAGACUUGGTT
Dicer1 siRNA（Homo sapiens ）	GCUUGAAGCAGCUCUGGATT	UCCAGAGCUGCUUCAAGCTT

Supplementary Table 3. Cloning primers

pri-miR-29b1 expression	Forward primer	CCGGAATTCCCCTTGCCTCTAAATGAT
	Reverse primer	CGCGGATCCACAAGAAGAAAGTCTGTTCA
pri-miR-29b2 expression	Forward primer	CCGGAATTCATTTGTAGTGACTGGTGTG
	Reverse primer	CGCGGATCCTGATGGCTGCTAGGAGTC
pri-miR-30d expression	Forward primer	CCGGAATTCCTCATGTAGGATATCAGGGT
	Reverse primer	CGCGGATCCAATAGGCGGTGACACTTT
LOXL4 expression	Forward primer	ATGGCGTGGTCCCCACC
	Reverse primer	TCAGATGAGGTTGTTCCTG
miR-29b1 promoter clone	Forward primer	CTTTTGTACTTCAGTCACTG
	Reverse primer	CTTCATAATGCTCTCTTACA
miR-29b2 promoter clone	Forward primer	CCCTCATTTGACAGGATT
	Reverse primer	AAGAGGATCTCAATGAAGA
miR-30d promoter clone	Forward primer	AAAAATGGTAAACCTTTAGCT
	Reverse primer	GACTTTCTGAACAAGAAAT
LOXL4-3’UTR clone	Forward primer	AGCTGTCACTGCACACTC
	Reverse primer	CAGAGTGTGAGAGGTGAG
mut-LOXL4-3’UTR mutagenesis	Forward primer	CTAAGTCCACGATTGCAAATGTCTTGGAGGAGTAT
	Reverse primer	TTGCAATCGTGGACTTAGATGGGGGACTGGGCCAT
mut-LOXL4-3’UTR 331 mutagenesis	Forward primer	CTTAGGGTACGACTATGGCCCAGTCCCCCATCTAAG
	Reverse primer	AGTCGTACCCTAAGAAACTGAGAGCTCCTGAATCC

Supplementary Table 4. qRT-PCR primers

Nos2（Mus musculus ）	Forward primer	GCAGAGATTGGAGGCCTTGTG
	Reverse primer	GGGTTGTTGCTGAACTTCCAGTC
Tnf-α（Mus musculus ）	Forward primer	CAGGAGGGAGAACAGAAACTCCA
	Reverse primer	CCTGGTTGGCTGCTTGCTT
Arg1（Mus musculus ）	Forward primer	AGACAGCAGAGGAGGTGAAGAG
	Reverse primer	CGAAGCAAGCCAAGGTTAAAGC
Il-6（Mus musculus ）	Forward primer	CCACTTCACAAGTCGGAGGCTTA
	Reverse primer	GCAAGTGCATCATCGTTGTTCATAC
CD206（Mus musculus ）	Forward primer	AAACACAGACTGACCCTTCCC
	Reverse primer	GTTAGTGTACCGCACCCTCC
EZH2（Mus musculus ）	Forward primer	AGTGACTTGGATTTTCCAGCAC
	Reverse primer	AATTCTGTTGTAAGGGCGACC
LOXL4（Mus musculus ）	Forward primer	GCCAACGGACAGACCAGAG
	Reverse primer	CCAGGTCAAGGCTGACTCAAA
EZH2（Homo sapiens ）	Forward primer	AATCAGAGTACATGCGACTGAGA
	Reverse primer	GCTGTATCCTTCGCTGTTTCC
SUZ12（Homo sapiens ）	Forward primer	AGGCTGACCACGAGCTTTTC
	Reverse primer	GGTGCTATGAGATTCCGAGTTC
EED（Homo sapiens ）	Forward primer	GTGACGAGAACAGCAATCCAG
	Reverse primer	TATCAGGGCGTTCAGTGTTTG
LOXL1（Homo sapiens ）	Forward primer	GGCTGCTATGACACCTACAATG
	Reverse primer	GTAGTGAATGTTGCATCTCACCA
LOXL3（Homo sapiens ）	Forward primer	TGGAGTTCTATCGTGCCAATGA
	Reverse primer	CCTGAGGCTTCGACTGTTGT
LOXL4（Homo sapiens ）	Forward primer	CTGGGCACCACTAAGCTCC
	Reverse primer	CTCCTGGATAGCAAAGTTGTCAT
Dicer1（Homo sapiens ）	Forward primer	AAAATTGTCCATCATGTCCTCGC
	Reverse primer	CCACCAGGTCAGTTGCAGTT
miR-29b1 for ChIP	Forward primer	GCTTTGTCCTATTTGCATGT
	Reverse primer	CCAGACAAAGGTTCAGCTT
miR-29b2 for ChIP	Forward primer	GCAGGGCAGTACAAATGTA
	Reverse primer	TCTACTTGTGATATGACACAG
miR-30d for ChIP	Forward primer	AGCAGGCATCCATGAAATGT
	Reverse primer	AAGTGGTTCACCAAGTGCAA
